# Molecular imprinting for neurology: Materials, applications, and limitations

**DOI:** 10.1002/ibra.70009

**Published:** 2025-12-04

**Authors:** Xiaohan Ma, Yingqi Ma, Claudia Marino, Alessandro Poma

**Affiliations:** ^1^ Division of Biomaterials and Tissue Engineering, UCL Eastman Dental Institute Royal Free Hospital, UCL Medical School London UK; ^2^ Department of Neurology, Sealy Institute for Drug Discovery and Mitchell Center for Neurodegenerative Diseases The University of Texas Medical Branch Galveston Texas USA

**Keywords:** brain, molecular imprinting, molecularly imprinted polymers (MIPs), neurology, therapeutics

## Abstract

Neurological disorders represent one of the most pressing challenges in contemporary medicine, requiring tools that enable early diagnosis, targeted treatment, and deeper mechanistic understanding. Conventional biological agents such as antibodies, though widely used, often face limitations related to stability, brain penetration, cost, and integration into real‐world platforms. Molecularly imprinted polymers (MIPs) have emerged as a promising synthetic alternative, capable of mimicking the molecular recognition functions of biological systems while offering enhanced robustness and customisability. MIPs provide key advantages, including high thermal and chemical stability, reusability, and design flexibility, making them especially attractive for neurological applications. In this review, we begin by presenting an overview of the main MIP formats applied in neurology, detailing their preparation, characterisation, and application‐relevant advantages and limitations. We then explore the most actively investigated areas of MIP use in neurological diagnostics, research, and therapy, with a particular focus on: (i) nerve agents, (ii) neurotransmitters, and biomarkers, and (iii) drug development, drug delivery, and direct biological activity. Finally, we discuss the key challenges that currently hinder the clinical translation of MIPs in neurology, including poor biodegradability, in vivo biocompatibility concerns, and scalability, along with emerging strategies aimed at overcoming these barriers. We hope this analysis will serve as a useful reference for neuroscientists seeking novel material‐based tools, as well as for materials scientists aiming to develop neurological applications of molecular imprinting.

## Graphical abstract

Molecularly imprinted materials: diagnostic, therapeutic and research applications in neurology. Molecularly imprinted materials offer high specificity and affinity for target molecules in neurological applications. This review highlights their synthesis, characterisation, and use in diagnostics, research and therapeutics. It addresses design principles, biocompatibility, and integration into devices, emphasising their potential to transform neurological interventions.

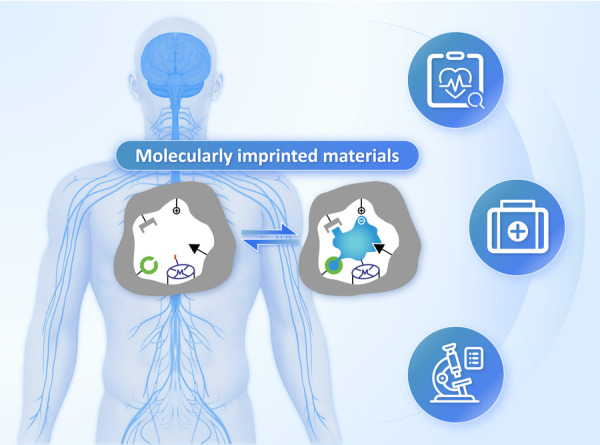

## 1 NEUROLOGICAL DISORDERS AND DAMAGE: AN OVERVIEW OF DIAGNOSIS, TREATMENT AND MANAGEMENT

Neurology, the branch of medicine focused on the study and treatment of disorders affecting the nervous system, plays a pivotal role in understanding some of the most complex and debilitating conditions affecting humans.^1^ The nervous system, comprising the brain, spinal cord, and peripheral nerves, is responsible for regulating and coordinating all bodily functions.^2^ Consequently, neurological disorders can have profound impacts on both physical and cognitive abilities.

Neurological disorders encompass a wide range of conditions, including neurodegenerative diseases like Alzheimer's (AD) and Parkinson's (PD), neuroinflammatory diseases such as multiple sclerosis, cerebrovascular diseases like stroke, and neurodevelopmental disorders including autism and epilepsy. Each of these conditions presents unique challenges in terms of diagnosis, treatment, and management.^1^

Early and accurate diagnosis of neurological disorders is crucial for effective management and treatment; however, this remains a significant challenge due to the complex and often overlapping symptoms of many neurological conditions. Diagnostic strategies typically involve a combination of clinical evaluation, neuroimaging, electrophysiological studies, and laboratory tests.^1^ Clinical evaluation by a neurologist includes a thorough medical history and neurological examination to assess cognitive function, motor skills, sensory perception, and reflexes. Neuroimaging techniques such as magnetic resonance imaging (MRI) and computed tomography (CT) scans are indispensable tools in this process. Specifically, MRI provides high‐resolution images of brain structures and is instrumental in detecting abnormalities such as tumours, inflammation, and degenerative changes, while functional MRI (fMRI) and positron emission tomography (PET) scans offer insights into brain activity and metabolism, aiding in the diagnosis of conditions like epilepsy and dementia.^3^, ^4^ Electrophysiological studies, including electroencephalography (EEG) and electromyography (EMG), are also essential for diagnosing disorders related to electrical activity in the nervous system. EEG is particularly useful in epilepsy and sleep disorders, whereas EMG assesses muscle and nerve health in conditions such as amyotrophic lateral sclerosis (ALS) and peripheral neuropathies.^5^ Laboratory tests, including blood and cerebrospinal fluid (CSF) analyses, further provide valuable information about infections, autoimmune conditions, and metabolic disorders affecting the nervous system.^6^, ^7^ Moreover, the identification of specific biomarkers such as amyloid‐beta and tau proteins is an emerging research area, offering potential for early detection of AD even before the onset of clinical symptoms.^1^, ^8^

The treatment and management of neurological disorders likewise require a multifaceted approach that integrates pharmacological therapies, surgical interventions, lifestyle modifications, and supportive care. The overarching goals of therapy are to manage symptoms, slow disease progression, and improve quality of life. Pharmacological treatment remains central to neurological care: medications can alleviate symptoms, modify disease progression, and target underlying mechanisms. For instance, cholinesterase inhibitors and N‐methyl‐d‐aspartate (NMDA) receptor antagonists are used in AD to enhance neurotransmitter function and protect neurons, while dopamine (DA) replacement therapy with levodopa (l‐DOPA) markedly improves motor symptoms in PD.^9^, ^10^ In multiple sclerosis, immunomodulatory therapies such as interferon‐beta and monoclonal antibodies reduce inflammation and prevent relapses.^1^, ^11^ When pharmacological treatments are insufficient, surgical interventions become critical. Deep brain stimulation (DBS), for example, is employed in PD and essential tremor to modulate abnormal neural activity, and in epilepsy, surgical resection of epileptogenic tissue can be curative. Neurovascular procedures such as aneurysm clipping and carotid endarterectomy also play a crucial role in stroke prevention.^12^ In addition, lifestyle modifications (including diet, exercise, and cognitive training) offer complementary benefits, contributing to improved patient outcomes and long‐term management of neurological disorders.^1^, ^11^

Another extremely important aspect in relation to neurological damage and disorders is the exposure to nerve agents. These latter are highly toxic chemicals that interfere with the nervous system by inhibiting acetylcholinesterase, an enzyme essential for the breakdown of the neurotransmitter acetylcholine.^13^ This results in an accumulation of acetylcholine, leading to continuous stimulation of muscles, glands, and the central nervous system, which can be fatal if not treated promptly. Common warfare nerve agents include sarin, venomous agent X (VX), and tabun, but also organophosphate pesticides (e.g., parathion) or other industrial chemicals (e.g., tetraethyl pyrophosphate) can exhibit a similar neurotoxic action. Symptoms of exposure include muscle twitching, respiratory distress, convulsions, and loss of consciousness.^14^ Treatment of these types of poisoning involves the rapid administration of antidotes to counteract the effects of acetylcholine accumulation, such as atropine (an anticholinergic drug), which is used to block the action of acetylcholine at muscarinic receptors to alleviate symptoms such as bronchial constriction and excessive salivation. Other compounds (e.g., oximes) reactivate acetylcholinesterase by cleaving the bond formed between the enzyme and the nerve agent.^14^

The complexities of neurological disorders and the urgent need to improve early diagnosis and precise treatments underscore the importance of novel solutions. Advances in materials science and biotechnology offer exciting opportunities to develop tools that mimic biological specificity, potentially enabling breakthroughs in both diagnostics and therapeutics. Among these innovations, molecular imprinting stands out as a promising approach to address key obstacles such as the blood–brain barrier (BBB), the need for highly selective biomarkers, and the limited efficiency of current drug delivery systems.

In this context, the present review provides a comprehensive overview of the current state of molecularly imprinted materials in neurology. In the following sections, we discuss how these materials have been explored for three major application areas: (i) sensing and detection of neurological biomarkers, (ii) targeted therapeutic delivery across the BBB, and (iii) tools for preclinical research and neurotoxin deactivation. We have strived to highlight the current technological approaches, key challenges such as biocompatibility, scalability, and in vivo performance, as well as emerging strategies that aim to overcome these barriers. By bridging material science and neuroscience, this review emphasizes how molecularly imprinted materials may offer transformative potential in neurobiomedical research and therapy.

## 2 MOLECULARLY IMPRINTED MATERIALS: A USEFUL APPROACH IN SOLVING CHALLENGES IN NEUROLOGY

Molecularly imprinted polymers (MIPs) are synthetic materials designed with specific recognition sites for target molecules, emulating natural binding mechanisms in biological systems. These materials present a novel and promising avenue for neurological applications, particularly in diagnostics and therapeutics.^15^, ^16^ The molecular imprinting technology creates custom binding sites within a specific matrix, usually (but not limited to) a cross‐linked polymer matrix. These sites possess specific size, shape, and chemical functionality, enabling high‐affinity, specific recognition and rebinding of target molecules (“templates”), akin to antibodies and natural receptors recognising their ligands and antigens.^17^, ^18^, ^19^

**Figure 1:**
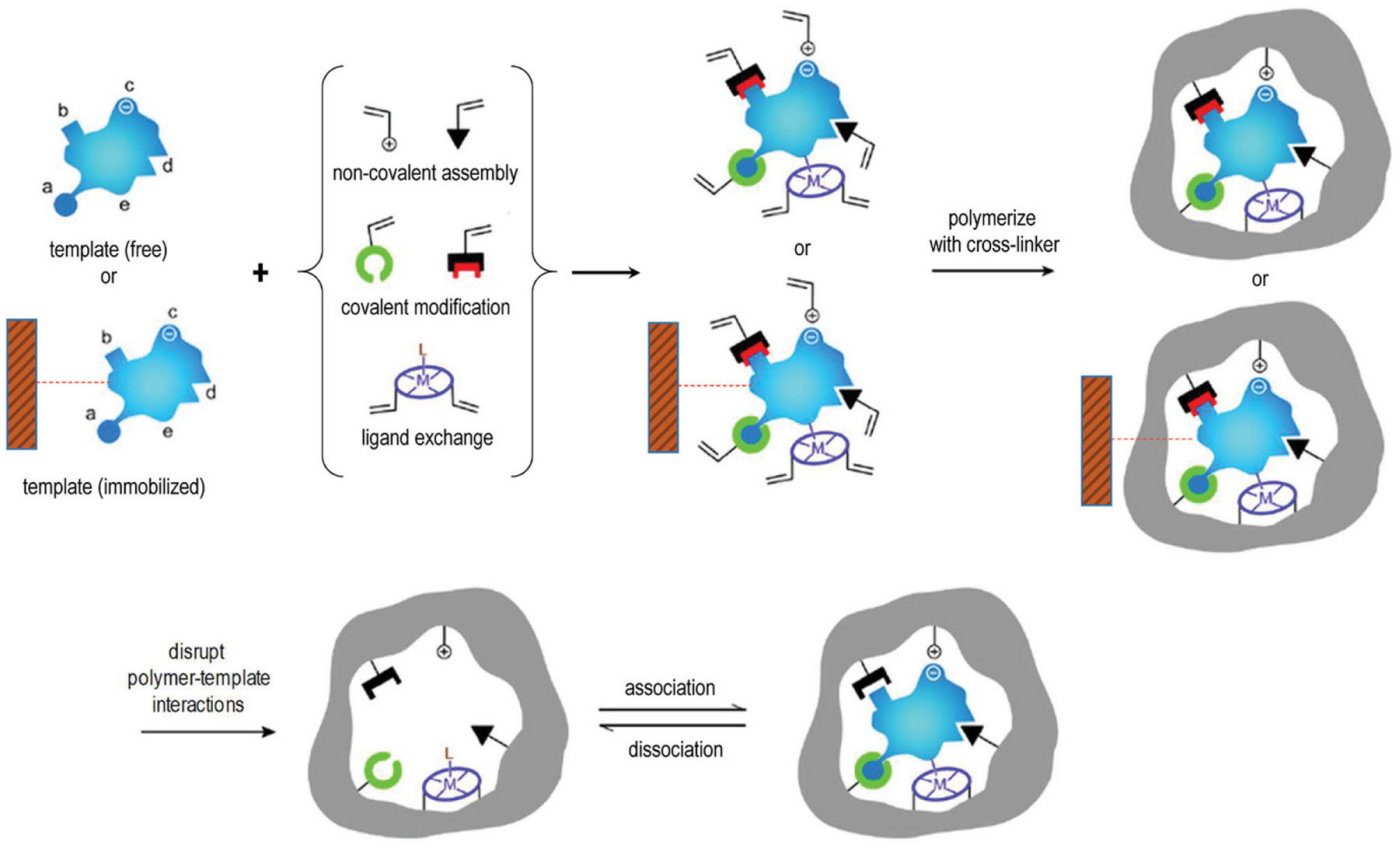
Scheme of the molecular imprinting process. Interactions between the template (free in solution or immobilised on a suitable solid support) and polymerisable groups are either covalent, noncovalent, or via co‐ordination with a metal forming a complex with suitable functional groups or structural elements of the template. Subsequent polymerisation (usually in the presence of a cross‐linker) develops a porous insoluble matrix containing the binding sites for the template. At this point, either the template is removed (if free), or alternatively the polymer is separated from the immobilised template exploiting suitable washing/elution conditions. In all cases, the target analyte can selectively rebind to the polymer into the sites formed by the template, or “imprints.” Adapted with permission from Patel et al.^19^

Binding site formation occurs throughout the imprinting process, which includes the pre‐polymerisation stage (where interactions between the template and functional monomers are established), the polymerisation and/or cross‐linking stage (during which the matrix is formed around the template), and the template removal stage (which reveals the specific binding cavities). Templates are typically removed through suitable washing steps. Alternatively, in some molecular imprinting strategies, solid‐phase synthesis is employed, immobilising the template on a supporting surface like glass, silicon, or iron oxide (Figure 1).^20^, ^21^, ^22^

Although the main role in the interaction with the template is played by the functional moieties present in MIPs,^22^, ^23^, ^24^ the overall affinity arises from the target and binding site shape, physicochemical interactions, as well as environmental factors (e.g., temperature, pH, ionic strength).^25^, ^26^, ^27^, ^28^ This differs from ligand‐based binding materials, where known binding moieties are incorporated without target presence during preparation. In these cases, binding relies solely on binder‐target interaction (e.g., biotin‐streptavidin), without specific binding site creation.^29^, ^30^, ^31^, ^32^, ^33^, ^34^

The resulting MIPs normally exhibit high stability and robustness, even under harsh conditions, making them cost‐effective alternatives to natural and animal‐derived reagents like antibodies. Over the past five decades, MIPs in particular have been utilised in various applications, including solid‐phase extraction (SPE), chromatographic separation, peptide and biomolecule recognition, hazardous waste capture, drug delivery, sensing, and even pseudo‐immunotherapy for cancer or other diseases.^19^, ^35^, ^36^, ^37^, ^38^, ^39^

MIPs have an exceptional versatility and a capacity for seamless integration with other platforms and materials. Therefore, it is not surprising that MIPs represent a highly adaptable class of biomaterials. Their properties underpin the easy translation of MIP‐based approaches into a broad spectrum of therapeutic interventions, including those targeting the central nervous system.^18^, ^23^, ^35^, ^40^, ^41^, ^42^

Additionally, MIPs could be utilised for targeted drug delivery and controlled release systems. The ability of MIP nanoparticles (MIP NPs), for example, to selectively bind and release therapeutic agents in response to specific triggers, makes them ideal candidates for crossing the BBB and delivering drugs directly to the brain.^18^, ^37^, ^38^, ^39^ Compared to their larger‐scale counterparts (e.g., MIP microparticles),^43^ MIP NPs benefit from attributes that are particularly advantageous in neurological applications, such as higher surface‐to‐volume ratio, faster binding kinetics, and improved tissue penetration. Unlike natural receptors (e.g., antibodies or enzymes), they offer higher stability and longer shelf life; compared to synthetic receptors such as aptamers, they provide greater resistance to enzymatic degradation and more flexible material design.^18^, ^22^, ^39^ This could significantly enhance the efficacy and reduce the side effects of treatments for neurological disorders. Alternatively, MIPs could be used towards neuroprotection in response to oxidative stress or inflammatory signals, or as synthetic nerve antidotes with improved activity.

Significant challenges, however, lie in adapting MIP platforms to the unique demands of neurological applications.^19^, ^37^, ^38^, ^39^ The brain presents a highly selective environment due to the presence of the BBB, the dynamic composition of extracellular fluids, and the tight regulation of neurotransmitter levels. While MIPs have demonstrated promising selectivity for small molecules such as DA, serotonin (5‐HT), and acetylcholine in vitro, translating this recognition to clinically relevant matrices remains difficult. Similarly, the development of MIP‐based carriers for brain‐targeted drug delivery requires careful consideration of size, surface chemistry, and degradability to ensure effective penetration and minimal immune activation. These neuro‐specific constraints necessitate a shift from generic polymer design to tailored materials that can operate reliably under physiological conditions. The following sections examine how different MIP formats and how they have been adapted for sensing, therapeutic delivery, and even direct modulation of neural function.

## 3 TYPES OF MATERIALS, PREPARATION AND CHARACTERISATION

### 3.1 Bulk MIPs

Classically, MIPs have been produced as bulk polymers. These types of materials are the results of a free radical polymerisation process in the presence of high concentrations of monomers and cross‐linkers (which are historically encouraged to achieve a robust and successful imprinting of the target species) and brought to completion, usually thermally or via ultraviolet (UV) irradiation. The products are obtained primarily as large monolithic blocks, that are routinely ground and dry‐ or wet‐sieved into micrometre‐sized particles for further processing and application. The removal of the template is normally performed via washings and/or Soxhlet extraction.

Despite its traditional approach, bulk imprinting remains straightforward and accessible due to its simplicity and lack of specialised equipment. For instance, bulk‐produced MIP powders can be easily packed into SPE cartridges and high‐performance liquid chromatography (HPLC) columns for dedicated adsorption, purification, isolation, and analysis purposes, including the detection of nerve agents and neuro‐contaminants.^44^, ^45^, ^46^, ^47^, ^48^, ^49^, ^50^, ^51^, ^52^ In some cases, directly monolithic columns are generated e.g., for capillary electrophoresis (CE) or even HPLC and SPE.^53^, ^54^, ^55^ It should be noted, though, that for these supports and applications, an adequate optimisation of the porosity of the obtained monolith should be performed to avoid any issues with backpressure or elution rate.^54^

Furthermore, these MIPs can be integrated onto sensors, such as potentiometric,^56^ luminescence‐based,^57^, ^58^, ^59^, ^60^, ^61^, ^62^ voltammetric,^63^ impedometric,^64^, ^65^ surface plasmon resonance (SPR) or quartz crystal microgravimetry (QCM) sensors.^35^, ^36^, ^40^ This approach, however, might result in uneven surface coating (either due to the polydispersity of the MIP powder obtained after the sieving process, or potential aggregation phenomena required for the immobilisation). Moreover, usually a curable dispersion matrix is required (e.g., an additional polymer) which might cause interference during the measurement process.^56^, ^62^, ^64^ To obviate these issues, an alternative method involves partial polymerisation in bulk, followed by completion of the polymerisation after coating the sensor surface (e.g., by dipping or spin‐coating).^57^ This strategy has the advantage of not requiring an additional dispersion medium to attach the MIP to the sensor. Nonetheless, depending on the polymer composition and the sensor mechanism, careful optimisation is required to ensure compatibility between the MIP and the sensing layer for transducing the binding event into a signal. As an example, a switch to more controlled polymerisation techniques such as atom transfer radical polymerisation (ATRP) or reversible addition‐fragmentation chain transfer polymerisation (RAFT) might be required to maximise compatibility with certain exotic monomers necessary to ensure adequate detection and transduction. Nonetheless, this could be counterproductive in some cases (e.g., reduction in matrix porosity).^61^

While simple, bulk polymers may exhibit a wide range of affinities within the created binding sites due to suboptimal binding cavities and a lack of a selection process. Additionally, template molecules may become entrapped within the MIP structure, potentially leaking out and posing issues for applications requiring low‐limit detection and sensing.^59^

### 3.2 MIP films, membranes and hydrogels

Although bulk polymers can and have been used extensively, even for more specialised applications such as drug delivery,^66^ drug discovery^67^ and even catalysis,^68^, ^69^, ^70^ other formats are more practical and better performing, particularly when considering the significant differences in production processes, affinity distribution and surface areas. Membrane formats are better suited for purification and separation applications,^71^, ^72^, ^73^ whilst sensing elements, in particular, benefit from being based on a MIP film.^74^, ^75^, ^76^, ^77^ These formats more easily allow for using techniques such as electropolymerisation, solvent casting/spin‐coating coupled with photo‐ or thermal polymerisation, surface‐initiated polymerisation processes (e.g., ATRP or RAFT) or grafting, thus enabling fine‐tuning of the MIP layer's thickness, cross‐linking density and potentially introducing stimuli‐responsiveness.^63^, ^64^, ^78^, ^79^, ^80^, ^81^, ^82^, ^83^, ^84^, ^85^, ^86^, ^87^, ^88^, ^89^, ^90^, ^91^, ^92^, ^93^, ^94^, ^95^ This precision is particularly crucial for optimising reusability or continuous monitoring, ensuring sufficient target binding and easy removal for subsequent measurements/separations.^64^, ^86^ Furthermore, it is important to maintain the compatibility with the transduction element in the case of sensors (e.g., SPR).^84^ The creation of multilayer or nanopatterned architectures, with distinct compositions for transducer and sensing layers, is extremely relevant,^90^, ^96^, ^97^, ^98^, ^99^, ^100^ with the potential application of different types of polymerisation techniques (e.g., electropolymerisation coupled with photopolymerisation)^81^, ^85^, ^96^ and/or exotic monomers (even nanocomposites) or initiators.^82^, ^93^, ^101^, ^102^, ^103^ Alternatively, the surfaces onto which films are deposited can also be exploited to immobilise the target template and therefore support the imprinting process (e.g., by establishing a specific template orientation),^104^, ^105^, ^106^, ^107^ although depending on the immobilisation chemistry, it might be challenging to optimise the conditions for the removal of the target after the polymerisation without damaging the imprinted film.^106^, ^108^

Nevertheless, the above‐mentioned MIP architectures generally might be hindered by relatively low capacity or significant cross‐reactivity, which may limit their use in real‐world applications.^109^ Even if some of these issues might be addressed at the application development and optimisation stage,^94^, ^108^ they could still represent a major drawback. Unless the monomer composition chosen allows for a suitable regeneration of the MIP product, most probably a compromise would have to be accepted in terms of recognition performance loss and reusability of the material.^108^ In some cases, an increase in surface area has been attempted by physically shredding the film into smaller particles, but with limited success and incurring into the same problems discussed above for the bulk MIPs applied to sensing.^110^ Furthermore, some of these formats require the addition of a plasticiser or of specific preparation steps (e.g., phase‐inversion), which could interfere with the polymer formation and the imprinting process.^71^, ^74^ In certain cases, auxiliary membranes have been introduced to help reduce the interference from contaminants, but this approach could eventually result in higher costs as well as significantly prolonging the production times.^109^

Although hydrogels are not normally amongst the most suitable formats for molecular imprinting, due to their inherent molecular flexibility and swelling properties, one example has been reported in the literature, particularly for detoxification applications (vide infra).^111^ Nonetheless, the required complexity for the production process of this hydrogel might hinder its widespread adoption as well as negatively affect the reproducibility of its fabrication and performance. In some cases, composite formats such as hydrogels embedded with MIP microparticles have also been produced (for implantation post‐surgery).^112^ Separating the imprinting process from the generation of the hydrogel architecture could allow for a better integration of the imprinting properties into these formats.

### 3.3 MIP microparticles

Microspheres and microparticles are particularly relevant for drug delivery, separation/purification or even catalysis applications.^113^, ^114^, ^115^, ^116^, ^117^, ^118^ They can be prepared using various methods like precipitation polymerisation,^112^, ^115^, ^116^, ^119^, ^120^ emulsion polymerisation, suspension polymerisation,^113^, ^114^, ^117^ and microfluidics. These methods are potentially scalable and allow for core‐shell architectures to be obtained, where the core serves a different function than the imprinted layer, such as carbon (to increase conductivity) or noble metals (for detection/catalysis or magnetism for efficient separation).^113^, ^114^, ^115^, ^116^, ^118^, ^121^, ^122^ The core in certain instances can act as a macroinitiator for the polymerisation reaction (“grafting from” approach).^122^ Micron‐sized spherical particles can be easily handled, centrifuged or filtered, packed in SPE cartridges or HPLC columns for selective purification or analysis of target molecules.^117^, ^118^, ^120^ Nevertheless, the production parameters need to be optimised and might require the auxilium of additional components (e.g., a porogen in addition to a solvent) to ensure that the produced materials exhibit a sufficient surface area for the application required.^119^, ^120^ This in turn might complicate the production process and also exhibit a significant effect on the final costs, both in terms of synthesis and hazardous waste handling. Furthermore, in the case of core‐shell architectures, the nature of the core (and its size) or the post‐synthetic processing might significantly influence the resulting final product,^114^, ^115^, ^121^ ultimately hindering one of the main advantages of the microparticles format over the bulk polymers, namely the spherical morphology.

### 3.4 MIP NPs

Therapeutic applications represent a key area of interest for MIP NPs (vide infra), where their nanoscale dimensions offer distinct advantages over larger MIPs (e.g., MIP microparticles), such as enhanced molecular recognition efficiency and deeper tissue accessibility.^43^ Moreover, compared to natural and synthetic receptors, MIP NPs combine robustness under harsh conditions with lower production cost, although they often lack the conformational adaptability of biological systems.^123^ Despite these promising features, their broader clinical application remains limited mainly by concerns surrounding biocompatibility and biodegradability.^124^, ^125^ The polymers normally used for MIP production are not amongst the most biocompatible, and therefore a certain trend is involving the usage of silicon‐based MIP NPs.^126^, ^127^, ^128^, ^129^ A major advantage of the MIP NPs format, however, is its easy integration with other formats (e.g., microspheres, films, membranes, fibre mats, etc.),^19^, ^64^, ^130^, ^131^ which dramatically expands the potential applications whilst at the same time retaining the better recognition abilities and handling properties typical of the NPs format. Although some progress has been made towards more environmentally friendly molecular imprinting technology, significant work is still needed to balance the strong, robust bonds required for imprinting with the need for biodegradability.^125^ The usage of endogenous or biodegradable polymeric materials could help with that, and even core‐shell architectures are particularly useful, either when the mass‐transfer of the analyte represents an issue (e.g., protein imprinting) but also to introduce additional types of capacity within the NPs products, such as fluorescence or magnetic properties.^76^, ^127^, ^129^, ^132^, ^133^, ^134^, ^135^, ^136^ To improve biodegradability, in some cases, specific unusual cores can be exploited (e.g., starch‐based).^101^ MIP NPs systems, however, particularly if core‐shell, are not amongst the simplest to produce, particularly because it becomes challenging to tailor the right conditions to achieve good physicochemical properties as well as the molecular imprinting effect.^137^ Bulk polymerisation is inefficient and low‐yielding to produce nanosystems, and other strategies such as emulsion polymerisation are quite complex and cost‐ineffective in the long term (high energy use, complexity and costs per yield unit).^64^ Nevertheless, MIP NPs have been a major focus in recent molecular imprinting research.^17^ The introduction of solid‐phase synthesis strategies has enabled the simultaneous production and purification of MIP NPs, which closely resemble natural monoclonal antibodies in size and recognition performance.^18^, ^20^, ^21^, ^22^, ^23^, ^24^, ^25^, ^26^, ^27^, ^37^, ^40^, ^128^, ^131^, ^138^, ^139^, ^140^ Although this method is now relatively established, research groups are now exploring its fine optimisation (e.g., influence of the type of solid phase linker on the imprinting properties, modification of the type of solid phase, etc.).^140^, ^141^ Another recent trend to help with the usual disadvantages of MIPs (such as binding site affinity distribution and low adsorption) is to use mesoporous silicon NPs or metal‐organic frameworks (MOFs) as cores.^142^, ^143^, ^144^ MOFs are a relatively new type of porous materials with a three‐dimensional pore structure that have developed rapidly in recent years, whose surface area is large, porosity is high, structure is regular, and quality is controllable.^143^, ^144^ Nonetheless, the presence of certain heavy metals might represent a risk in the production and handling of these composite materials, as well as hindering their broader applications. Exotic monomers and cross‐linkers can also be used to aid with the imprinting or the biocompatibility/biodegradability.^76^, ^132^, ^142^, ^145^ In some cases, metals can be used as coordinating centres to increase the imprinting efficiency (e.g., cobalt),^146^ or even complex decorating strategies have been used to maximise the overall performance.^147^ In these cases, however, the chemical complexity to obtain the final product needs to be evaluated against the full cost of the process in terms of time and resources required.

Even nanotubes are used for molecular imprinting within the neurology area, although the synthetical complexity and hazardous chemical processing hinder the overall development of this architecture for these applications.^103^, ^148^

## 4 CURRENT APPLICATIONS OF MOLECULAR IMPRINTING IN NEUROLOGY

### 4.1 MIPs for nerve agent detection, analysis and removal

The application of MIPs towards nerve agents and their metabolites in particular is something that is extremely relevant, and many examples of MIPs have been published on the subject.^44^, ^45^, ^51^, ^56^, ^57^, ^58^, ^59^, ^60^, ^61^, ^68^, ^69^, ^70^, ^74^, ^75^, ^76^, ^77^, ^86^, ^88^, ^110^, ^111^, ^113^, ^119^, ^129^, ^137^, ^139^, ^145^, ^146^, ^149^, ^150^, ^151^ The majority of these examples relate to the development of MIP‐based sensors [in some cases with limits of detection (LoD) down to the parts per trillion (ppt)], whilst others are more specific towards the preconcentration and purification of samples for subsequent analysis (e.g., via HPLC‐MS), or even catalysis (e.g, towards detoxification or decontamination).^150^, ^152^, ^153^, ^154^, ^155^

The very first application explored in neurology using MIPs dates back to 1997, and has been the development of a luminescent sensor for the detection of nerve agents.^57^ Since then, Murray et al. have heavily developed this luminescent detection technology based on the spectral difference exhibited by a complex between Europium^III^ and the hydrolysis product of the targets, pinacolyl methyl phosphonate (PMP).^61^ Polymers with good sensitivity [LoDs in the low parts per billion (ppb) range] and high selectivity (no interference detected from chemical structural analogues) were obtained, although this was improved throughout the years thanks to the use of controlled polymerisation processes (e.g., RAFT) and combinatorial chemistry approaches.^61^, ^75^ Another potential issue is related to the detection method based on the hydrolysis product of the nerve agents, which might cause interference and false positive in the presence of certain types of compounds such as pesticides and insecticides.^57^, ^58^, ^59^, ^60^, ^61^, ^149^ For the same target, Malosse et al. developed an assay based on MIP microspheres and the competitive quenching of a fluorescent dye, although their data show limited differences in binding between MIP and nonimprinted polymers (NIPs), possibly due also to the choice of rebinding solvent (toluene, which is highly hydrophobic).^119^ The exhibited cross‐selectivity is not always a disadvantage, though, since in the case of SPE applications, it can be exploited to concentrate not only the nerve agent target but also its active metabolites/degradation products.^44^ Zhou et al. developed a truly elegant and simple potentiometric sensor for methylphosphonic acid (MPA), a degradation product of sarin, soman, VX, and so forth, based on an octadecyl silane layer, which exhibited remarkable selectivity considering the relatively simple hydrophobic/packing interactions at the base of the molecular imprinting process.^76^ However, the accuracy, precision and sensitivity were rather poor as a significant response was exhibited only in the range 10^−2^ to 10^−1^ M of MPA. Moreover, the sensor exhibited a loss in performance in response to a drop in pH, but this might be solved by relying on a different pH‐independent transduction mechanism.^77^ These results are in accordance with LeMoullec et al., who performed a head‐to‐head comparison of the same polymer imprinted in dichloromethane or acetonitrile, and showed that the polymer produced in acetonitrile was the only one exhibiting an imprinting effect for the template, therefore indicating that hydrophobic interactions might be dramatically important in the case of these type of targets.^45^

Prathish et al. improved on the potentiometric sensor of Zhou et al. by dispersing bulk MIP particles in 2‐nitrophenyloctyl ether (plasticiser) and embedding them into a polyvinyl chloride matrix. In this way, they managed to expand the linear response to the target whilst maintaining selectivity and reusability.^56^ The same authors attempted to create a monolithic MIP membrane in situ via a semi‐covalent imprinting approach to develop a potentiometric sensor for diethyl chlorophosphite (DCP). Although they were successful in performing the molecular imprinting and membrane formation at the same time as sensor construction, this process requires further optimisation to improve the sensitivity as well as selectivity for the target.^74^ On the same trend, Vergara et al. prepared a QCM sensor for PMP based on the electropolymerisation of thiophene, which allowed the authors to simultaneously perform and monitor the polymerisation, imprinting, film deposition and template removal. This strategy is extremely practical in terms of quality control and also tailoring of the sensing surface, but the selectivity of the sensor could benefit from improvement.^86^

In 2022, Yagmuroglu reported a potentiometric sensor for parathion based on MIP NPs. Although the performance was significantly superior to that of some of its bulk counterparts in terms of LoD and linear response range, the heavy dependence of the response from the pH of the environmental solution, as previously mentioned, was still present.^137^

In a 2018 study, Roy et al. confirmed that ^31^P^24^NMR represents a useful aid to optimise the imprinting conditions of nerve agents and their metabolites by screening the functional monomer, cross‐linker, porogen and stability of pre‐polymerisation complex before and during polymerisation.^51^ Even computational studies have been exploited, and resulted in the successful generation of a chitosan‐based MIP to be used as a filling agent in gas masks or gas filters, and exhibiting a trapping capability towards the template dimethyl methylphosphonate (DMMP) far superior (50‐fold) to the routinely used activated charcoal.^110^, ^151^ One drawback of this study is represented by the selectivity testing, since the authors tested the selectivity in the presence of structurally unrelated compounds (as could be found in an explosive mixture in a terrorist attack), whilst it would have been interesting to assess the selectivity in presence of the template structural analogues.

An especially attractive application area of MIPs for nerve agents and other organophosphates is their catalytic degradation for chemical weapons decommissioning, counteracting nerve agent attacks, and protecting against organophosphate pesticide poisoning. Say et al. were amongst the first to propose phosphotriesterase‐mimicking surface imprinted MIP microbeads for selective organophosphate hydrolysis by *N*‐methacryloyl–histidine–copper(II) [MAH–Cu(II)] as a new metal–chelating monomer. Although the polymer exhibited specific catalytic activity (which was retained even after multiple uses), this was heavily dependent on the pH, which might represent an issue depending on the envisaged application.^113^ The same authors later on used a similar approach based on a metal chelating monomer [this time chitosan–Cd(II)] for the recognition and preconcentration of dimethoate via SPE, although their results also in this case seem to point to the need for optimisation due to the dependence of the performance on the pH of the solution.^145^

Jorge et al. exploited a complex of [Co(cyclen)(OH)(OH_2_)]^2+^ incorporated into a MIP nanogel for nerve agent catalytic degradation, whilst Mohamed et al. presented a porphyrin‐based MIP with a Mn^3+^ core for the oxidation of both nerve agents and sulphur mustards.^69^, ^146^ Another interesting example has been reported by Pan et al. aimed at the effective rapid detoxification from organophosphates. Their polymers incorporated either a mononuclear zinc (II)‐picolinamine‐amidoxime (Zn‐PAAO) or an Ag(I) pyridine‐amidoxime functional module to mimic the organophosphorus hydrolase enzyme to achieve hydrolysis in minutes.^68^, ^70^ In all of the above cases, however, the imprinting was optimised for an analogue undergoing a similar reaction, and therefore it should be tested at least in controlled conditions on the proper templates if not in the field. Furthermore, in some instances, the chemical complexity and potential instability of the complexes could represent an issue in terms of catalyst turnover. In a recent development, though, the same group generated a hydrogel bearing this MIP Ag‐complex functionality including a zirconium‐based MOF network. This dual‐pronged approach resulted in the synchronous detoxification of two different types of organophosphorus agents, dimethyl 4‐nitrophenyl phosphate (DMNP) and parathion, ultimately displaying the promising potential of MIP/MOF hybrid enzyme‐like catalysts.^111^

**Figure 2:**
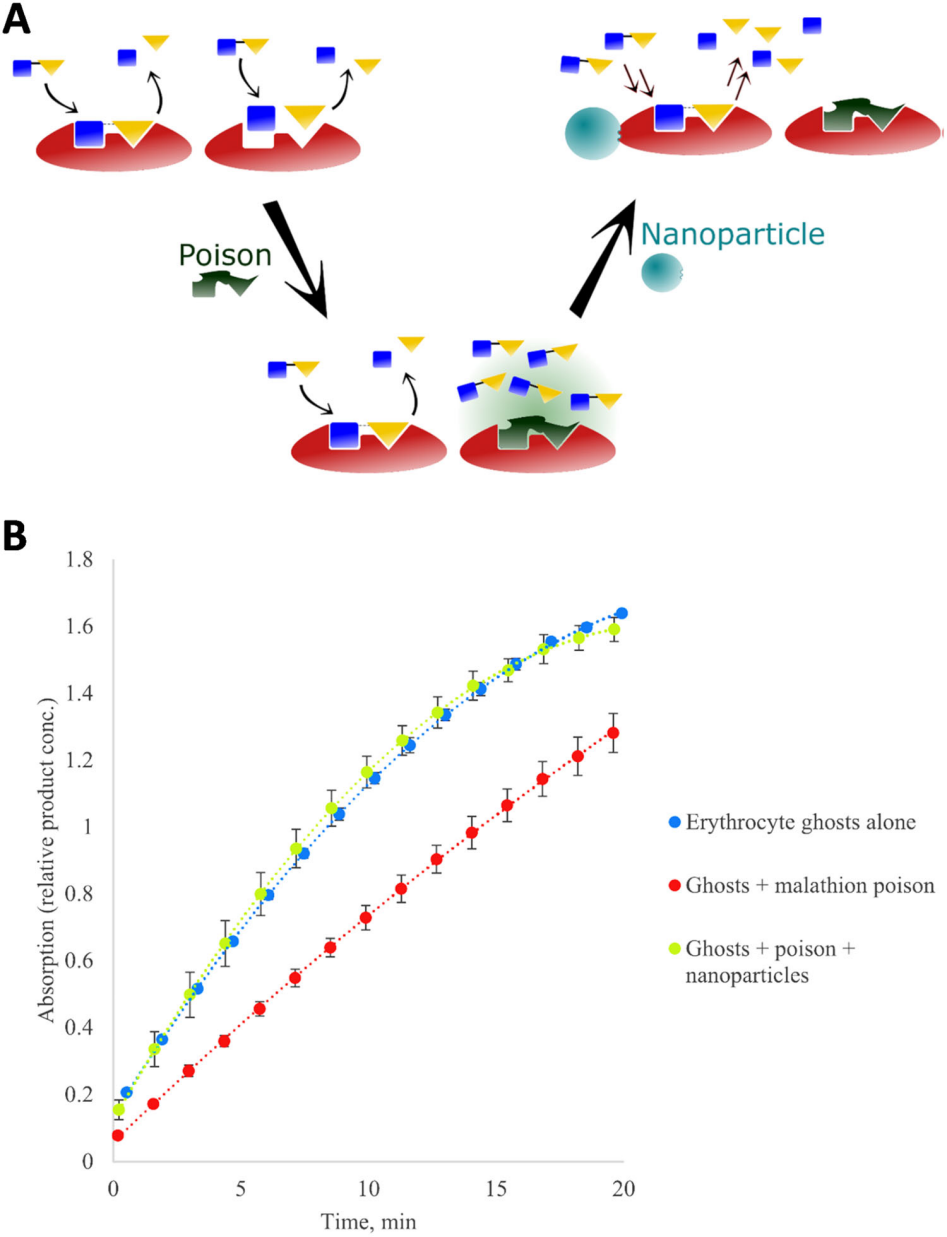
(A) Scheme of the proposed application of nanoparticles, in which the overall rate of acetylcholine hydrolysis may be maintained or restored in case of enzyme inhibition. (B) Demonstration of excessive nanoparticle administration for the maintenance of normal cholinesterase hydrolysis. Adapted with permission from Cowen et al.^139^

Thinking outside the box, instead of directly generating a synthetic catalyst to detoxify the nerve agents, Cowen et al. prepared MIP NPs focused on accelerating the natural cholinesterase activity leftover after the nerve agent action (Figure 2). Their results indicate an increase of acetylcholinesterase activity of >1.5‐fold, highlighting the therapeutic potential for this strategy.^139^

The principal studies discussed in this section are summarised in Table 1.

**Table 1:** Summary of representative MIP‐based systems for nerve agent detection, enrichment, or degradation.

Application type	Target molecule	Advantages	Disadvantages	Study (reference)
Luminescent sensor	Sarin, Soman hydrolysis products	High sensitivity (LoD ~125 ppt); ligand‐field luminescence with Eu³⁺; portable device miniaturisation feasible	Indirect detection through hydrolysis products; risk of false positives remains	[57]
Luminescent sensor	PMP	Sub‐ppb sensitivity; very high selectivity (no interference from similar organophosphates); RAFT polymerisation ensured stable Eu³⁺ complex incorporation	Still indirect detection; field validation is lacking	[61]
Fluorescent microsphere assay	PMP	Simple one‐step precipitation polymerisation; high surface area (up to 680 m²/g); high porosity with <2 nm pores; effective fluorescence‐based sensing using 4MU	Cross‐selectivity risk; limited validation in real complex samples	[119]
Potentiometric sensor	MPA	Rapid and specific recognition; high specificity, selectivity, stability, and fast response with ITO transducer	Strongly pH‐dependent response; validation in real matrices is limited	[76]
Potentiometric sensor	MPA	Wide linear range (5 × 10⁻⁸–10⁻¹ M); very low detection limit (5 × 10⁻⁸ M); reusable PVC composite; high selectivity over analogues; stable and portable design	Bulk particle dispersion affects sensor uniformity; conditioning requirements for reproducibility	[56]
Potentiometric sensor	DCP	In‐situ monolithic membrane; semicovalent imprinting improves uniformity; wide concentration range (10⁻⁶–10⁻² M)	Detection limit relatively high (LoD ~10⁻⁶ M); selectivity and sensitivity still require optimisation; response pH‐dependent	[74]
Potentiometric sensor	Parathion	Nanoparticle format; LoD 1.86 × 10⁻⁸ M; wide linear range (10⁻⁸–10⁻⁴ M); fast response (~20 s); validated in tap and lake water	Performance strongly pH‐dependent	[137]
Solvent comparison (MIP‐SPE)	Ethyl methylphosphonic acid (EMPA)	Good selectivity with TRIM‐based polymer in ACN; applicable to soil extract cleanup; recovery up to 93%; capacity ~97 µg/g	Sensitivity is limited compared to advanced sensors; extraction efficiency depends on solvent system (ACN vs. DCM)	[45]
QCM sensor	PMP	Electropolymerised polythiophene film; high sensitivity and selectivity; in situ EC‐QCM monitoring; LoD ~60 μM; good linearity in 60–200 μM range	Limited sensitivity for trace detection; partial cross‐reactivity; limited reusability	[86]
Computational/NMR optimisation	MPA (via DBMP derivative)	^31^P{^1^H} NMR enables rational design; screening of monomer, crosslinker and porogen; improved selectivity; reduced trial‐and‐error	Proof‐of‐concept only; limited validation; moderate adsorption capacity	[51]
Adsorption/SPE	DMMP	Chitosan‐based MIP: ~50‐fold higher adsorption capacity versus activated charcoal; strong binding and selectivity; maintained performance in the presence of common interferents (acetone, ethanol, isopropanol, ammonia, acetic acid)	Limited reusability due to MeOH extraction damage; performance against close structural analogues not fully assessed	[110]
Catalytic degradation	Paraoxon (nerve agent simulant)	MAH–Cu(II) surface‐imprinted microbeads mimic phosphotriesterase; simple and inexpensive preparation; reusable with only ~17% activity loss after several cycles; relative catalytic efficiency ~40‐fold higher vs NIP	Strong pH dependence (optimum pH 9.0); turnover is still much lower compared to natural enzyme	[113]
Adsorption/SPE	Dimethoate	Chitosan–Cd(II) MIP with ligand‐exchange strategy; very high adsorption capacity (up to ~720 mg/g); fast binding equilibrium (~35 min); selective recognition against other organophosphate esters (e.g., paraoxon, parathion)	Adsorption performance is strongly pH‐dependent; slower equilibrium observed in some formulations (up to ~120 min); optimisation still required	[145]
Catalytic hydrolysis	4‐nitrophenyl phosphate (NPP, nerve agent simulant)	First example of Co(cyclen)‐imprinted nanogels; low catalyst loading (15%) with good turnover; imprinting enhances catalytic activity; carbonate template >50% higher efficiency than phosphonate	Long‐term catalyst stability and reusability have not been validated; mechanism under different templates has not been fully clarified; tested only on model systems	[146]
Catalytic oxidative detoxification	Sulfur mustard (HD simulant) & VX simulants	Mn–porphyrin MIP catalyst; selective oxidation of sulfides to nontoxic sulfoxides; regio‐controlled P–S cleavage of VX; recyclable (≥3 cycles)	Catalyst still sensitive to H₂O₂; possible degradation of porphyrin under prolonged oxidative conditions	[69]
Catalytic degradation	Ethyl‐parathion, Methyl‐parathion, Fenitrothion	Ag(I)‐amidoxime MIPCPs; hydrolysis rate constant up to 1.2 × 10⁴‐fold faster than self‐hydrolysis; porous cross‐linked structure enhances catalytic activity; recyclable	Requires alkaline pH (≈9); relatively complex synthesis; substrate selectivity limited	[70]
Neuroprotection	Acetylcholinesterase (AChE)	Restored AChE activity in OP‐inhibited cells; accelerated cholinesterase activity to ~1.6‐fold; potential broad‐spectrum therapeutic strategy	No in vivo validation or biodistribution data; demonstrated only in cell membrane preparations	[139]

Abbreviations: 4MU, 4‐methylumbelliferone; AChE, acetylcholinesterase; ACN, acetonitrile; Ag(I), silver(I) ion; Co(cyclen), cobalt–cyclen complex; DBMP, dibutyl methylphosphonate derivative; DCM, dichloromethane; DCP, dichloropropyl phosphate; DMMP, dimethyl methylphosphonate; EC‐QCM, electrochemical quartz crystal microbalance; EMPA, ethyl methylphosphonic acid; Eu³⁺, europium(III) ion; HD, sulfur mustard; ITO, indium tin oxide; LoD, limit of detection; MAH, methacryloyl histidine; MeOH, methanol; MIPCPs, molecularly imprinted polymer catalytic particles; Mn–porphyrin, manganese porphyrin complex; MPA, methylphosphonic acid; NIP, nonimprinted polymer; NMR, nuclear magnetic resonance; NPP, 4‐nitrophenyl phosphate; OP, organophosphate; PMP, pinacolyl methyl phosphonate; ppb, parts per billion; PVC, polyvinyl chloride; QCM, quartz crystal microbalance; RAFT, reversible addition–fragmentation chain transfer; SPE, solid‐phase extraction; TRIM, trimethylolpropane trimethacrylate; VX, venomous agent X.

Taken together, these works can be broadly categorised into four main innovation areas. A first focus is on potentiometric and electrochemical sensors, exploring different matrix compositions (e.g., polyvinyl chloride [PVC] membranes, octadecyl silane layers, plasticiser‐dispersed MIPs) and recognition mechanisms to enhance selectivity and sensitivity toward nerve agent degradation products. A second focus is on efforts using electropolymerised thin films and QCM‐based platforms (e.g., polythiophene) enabled more reproducible and integrated sensor architectures with improved control over film morphology and signal stability. Third, multiple studies proposed MIPs as enzyme mimics or catalytic scaffolds, incorporating metal‐chelating monomers to degrade nerve agents or pesticides under physiological conditions, although many remain pH‐sensitive and require optimisation. Finally, hybrid MIP‐based systems, including MOF‐integrated composites and hydrogel platforms, represent an emerging frontier aimed at enhancing target accessibility, response dynamics, and dual‐functionality (e.g., detoxification and sensing). This diversity underscores the versatility of MIPs in the context of chemical threat mitigation, while also highlighting the persistent need for comparative benchmarking and in‐field validation.

While significant progress has been made in developing MIP‐based systems for nerve agent detection, analysis, and catalytic detoxification, however, several persistent limitations continue to hinder their transition from laboratory proof‐of‐concept to real‐world implementation. Most reported systems are evaluated under simplified or idealised conditions, often employing surrogate templates and lacking direct testing in complex biological or environmental matrices, which raises concerns about their selectivity and stability in practical scenarios. Moreover, many MIPs exhibit highly cross‐linked and rigid binding sites, which enhance selectivity under controlled laboratory conditions but can limit binding performance when the target molecules undergo structural or charge changes in complex environments such as those with varying pH or ionic strength. This lack of adaptability may reduce the sensor's accuracy and reliability in real‐world applications.^156^ Recent work on electropolymerised MIP thin films has demonstrated superior control over film thickness, morphology, and reproducibility, leading to improved signal stability and stronger integration with transducer surfaces.^157^ Similarly, hybrid materials such as MIP–MOF composites and stimuli‐responsive polymer architectures have proven effective in enhancing template accessibility, adaptive binding behaviour, and catalytic efficiency under variable conditions.^158^ To facilitate real‐world deployment, future research should prioritise evaluating MIP systems using real biological or environmental samples, including structurally related interferents rather than only unrelated controls. In addition, reproducibility should be assessed across different fabrication batches and sensor platforms to identify failure points and design limitations. Hybrid integration strategies, such as coupling MIPs with porous or adaptive matrices, also require validation under dynamic chemical conditions to ensure long‐term operational stability. These practical considerations form the basis for translational pathways discussed further in Section 5.

### 4.2 MIPs for neurotransmitters and biomarkers

An extremely important area of application of MIPs is for the analysis of neurotransmitters and biomarkers from complex tissues.^159^, ^160^, ^161^

#### 4.2.1 Sensors and assays

In addition to being a widely accepted model template in molecular imprinting due to its chemical nature, ease of access and electroactivity (as it can be oxidised on carbon electrodes), DA is a neurotransmitter of analytical clinical relevance, since often patients suffering from PD show DA variation from 1.22 ± 1.81 ng/mL to almost complete depletion.^162^, ^163^ One of the first examples of DA sensors was developed by Kan et al. by exploiting a composite of multiwalled carbon nanotubes (MWNTs) and MIPs.^148^ The electrochemical sensor could recognise DA selectively from ascorbic acids (AA) and noradrenaline, with an extended linear detection range. However, the modification of MWNTs to graft the MIP layer relies on treatment with strong acids, which might represent a health and environmental hazard. Since then, a plethora of sensors and assays have been developed for DA detection, relying on gold composites,^78^, ^79^ exotic zwitterionic polymers [poly(melamine‐*co*‐chloranil)],^63^ sol–gel derived ceramic‐MWNTs with a grafted MIP layer,^85^ electropolymerisation of *o‐*phenylenediamine and resorcinol,^83^ graphene sheets (with or without Congo red) MIPs,^121^, ^164^ electrochemical QCM with starch NPs‐reduced graphene oxide (RGO) nanocomposites.^101^ Even covalent approaches have been used, although with limited sensitivity.^62^

In an effort to contain the costs and dramatically simplify the production process, Huang et al. relied on an already formed polymer [poly(ethylene‐*co‐*vinyl alcohol)] as an imprinting layer, achieving relatively good selectivity for the target.^80^ It needs to be noted, though, that for certain sensing mechanisms, an intermediate electron transfer mediator needs to be included in the design and preparation, which could hinder the molecular imprinting process.^78^, ^79^ Lakshmi et al. sought to address this problem through the combination of a catalytic MIP (capable of oxidising the catechol template) with an electrically conducting polymer.^81^ The author's rationale was to generate a network of “molecular wires” to assist in the conduction of electrons from the active sites within the MIP to the electrode surface. Detection of catechol by the hybrid‐MIP sensor was found to be specific, with a 228 nM LoD. Catechol and DA were detected by the sensor, whereas analogues and interferents had minimal effect on the detection. However, significant synthetic efforts were required to generate both the polymers and the initiators required to achieve such a complex architecture.

To introduce an “internal reference signal” in the sensor and therefore increase the selectivity, Yang et al. developed an electrode with nanoporous gold to amplify the output signal and increase the surface to immobilise both polythionine (pThi) and DA‐specific MIPs.^90^ In this way, they achieved signals both for the oxidation of DA and pThi, since the oxidation peak currents of DA increased with increasing the concentration of DA, while the peak currents of pThi decreased simultaneously. Thanks to the specificity provided by the MIPs and the built‐in correction deriving from pThi, the fabricated sensor showed excellent performance in view of selectivity and reproducibility, although the LoD could benefit from improvement.

Gómez‐Caballero et al. were the first ones to pave the way towards the usage of porphyrin‐based monomers, since the oxidation of amino groups present in the structure of these monomers favours the electrochemical synthesis of an extremely stable polyporphyrin polymer with phenazine‐like structures (similar to those formed during the electrosynthesis of aniline).^82^ The developed sensor was successfully used for the determination of DA in brain tissue samples without exhibiting any interferences from the other neurotransmitters or substances.

Aiming at improving the LoD, Li et al. were able to develop a ultrasensitive DA sensor based on Au‐Ag alloy metallic microrods with an electropolymerised MIP layer, capable of detecting pM concentrations in serum and brain samples, although significant optimisation of the pH of the polymerisation process and the monomer/template ratio had to be carried out.^89^

With reusability and multiplexing in mind, Mintz‐Hemed et al. performed seminal work on chemical sensor platforms for neurotransmitters. First, they developed a fully reversible DA MIP sensor based on electrostatic repulsion, where the regeneration was guided by applying a small electrical potential. With a LoD of 760 pM maintained even after 30 sensing‐release cycles, these sensors could repeatedly quantify DA released from PC‐12 cells in vitro, demonstrating remarkable performance in complex biological environments. This strategy was successfully extended to a multiplexing system in a microelectrode array device integrated with a high‐density microwire bundle.^64^, ^65^

Instead of pursuing an electrochemical sensing platform, Dutta et al. developed a MIP SPR sensor for DA via the electrochemical polymerisation of *p*‐aminostyrene, whilst Pathak et al. performed the surface imprinting of DA in polypyrrole (PPy) over MWNTs and with nafion (a cation exchange polymer) as a membrane over imprinted sites to reduce the interference from anionic analytes (like AA and uric acid [UA] at physiological pH). The SPR analysis using both sensors allowed to achieve excellent sensitivity levels (down to pM concentrations) and high selectivity and regeneration capacity.^84^, ^109^ However, in both cases, no real samples were tested, so the performance in complex matrices could not be assessed.

Looking at a more established analytical strategy, Yoshimi et al. were among the first one to propose a fluorescent assay for DA and 5‐HT based on solid‐phase imprinted MIP NPs, generating interesting pilot data for the potential exploitation of these types of assays for the quantification of neurotransmitters, and at the same time providing significant information regarding the optimisation of solid‐phase synthesis based on the length of the spacer used for the immobilisation of the target.^140^ A similar approach and assay was also used by Zhang et al. for the rapid detection of brain natriuretic peptide (BNP), reaching a LoD of 0.208 pg/mL and success in the laboratory‐based analysis of over 150 clinical real serum samples.^141^ Afshar et al. developed a more complex fluorescent probe based on a Zr‐MOF encapsulated into a MIP (MOF@MIP) imprinted for melatonin (MLT), a hormone related to the regulation of brain functions and directly related to sleep quality. The probe allowed to detect trace amounts of MLT in real samples including grape, cherry, and sour cherry juice, with a LoD of 0.18 ng/mL.^144^ Nevertheless, it needs to be highlighted that fluorescent assays would still most likely be poorly compatible with a point‐of‐care scenario, ultimately limiting their application to a proper laboratory environment.

Adrenaline is another neurotransmitter of interest from a diagnostic perspective. Depending on the stress level, the adrenaline concentration range in human fluids is 0.015–40 μM.^165^ As such, elevated adrenaline levels could represent a useful biomarker for anxiety, depression, and posttraumatic stress disorder.^165^ These mental disorders have been increasing rapidly in society across all age groups, therefore, sensors that could monitor adrenaline in noninvasive biological fluids such as saliva or sweat could be useful for personalised self‐diagnosis and monitoring in both humans and animals. Yu et al. developed a capacitive sensor using a layer‐by‐layer (LbL) assembly strategy on a polydimethylsiloxane substrate, where they deposited carbon nanotube‐cellulose nanocrystals nanofilms (first layer), and adrenaline MIP poly(aniline/phenylboronic acid) moieties (second layer). The sensor exhibited an effective performance with high sensitivity and selectivity in real zebrafish brain samples (linear response between 0.001 and 100 μM and LoD of 0.001 μM) with a minimal sample consumption, although the reproducibility of the measurements required improvement.^96^

l‐Tryptophan (TRP) is a major precursor for numerous neurotransmitters and hormones (5‐HT, melatonin, vitamin B3, auxins, adrenaline, DA, and melanine). It exhibits some adverse effects like the production of toxic substances in the brain, leading to delusions, hallucinations, schizophrenia, PD and AD. Due to its importance as well as toxic effects, there is an increased interest in the accurate determination of TRP, and the group of Mani et al. contributed to this study avenue by developing a silver decorated silanized graphene oxide (silanised Ag‐GO) MIP electrochemical sensor for the selective and specific detection of TRP at sub‐nanomolar levels, even in blood serum samples, highlighting its potential for clinical applications.^95^

Glycine is widely distributed in the nervous system, playing key roles as a neurotransmitter and in the development and quality of our skeletal muscles, tissues and structural integrity. Considering the need for a reliable measurement of glycine in trace amounts,^166^ Zeng et al. developed a MIP polyaniline carbon fibre electrode for the determination of l‐glycine in CSF, with an excellent agreement with more established analytical methods and speed of analysis within 5 min when tested on real CSF samples obtained from rats.^87^

A crucial analyte to quantify in newborns is the serum bilirubin, which at high concentrations can cause brain damage and even death, therefore, Akhoundian et al. developed a bilirubin MIP voltametric sensor using a simple sol‐gel method, with a LoD of 0.75 μM and good selectivity in the presence of interferents such as cholesterol or testosterone.^126^ Parnianchi et al. fabricated a MIP‐based electrochemical sensor to detect neonatal jaundice in saliva and serum samples exploiting the polymerisation of 2‐aminothiophenol for bilirubin detection, achieving much lower LoDs (nM to pM) and rapid detection ability, cost‐effectiveness, low sample consumption, high sensitivity and selectivity, and good stability. Most importantly, the sensor was used in real saliva and serum samples, with excellent performance.^103^, ^148^

The pathophysiological changes contributing to AD typically begin decades before any signs of cognitive dysfunction can be diagnosed. Emam et al. observed specific changes in exhaled breath composition with the onset of the disease and experimentally validated this change using a rat model combining the human Apolipoprotein E 4 (ApoE4), a major genetic risk factor for sporadic AD, with aging and Western diet.^167^ They therefore developed gas sensors fabricated from MIP‐graphene and engineered to react with alkanes and small fatty acids associated with lipid peroxidation (specifically butylated hydroxytoluene, pivalic acid and 2,3‐dimethylheptane), to aid in the diagnosis of mild cognitive impairment. With LoDs of the order of ppt, the sensors were tested against the breath of wild‐type and APOE4 male rats, confirming that only APOE4 rats, and not wild‐type controls, tested positive to several small hydrocarbons and presented with reduced functional coupling in hippocampal circuitry, ultimately highlighting that this analysis of volatile compounds could potentially be used for presymptomatic detection of AD.^97^ A more thorough assessment of the selectivity for different volatile species, though, should be performed, followed by a thorough testing in a clinical setting. Instead of looking at the volatile compounds, several other groups specifically aimed at detecting the amyloid‐beta protein, which is causative for AD. Amyloid peptide β_1‐42_ (Aβ‐42), a component of the amyloid plaques that characterise AD, has been used as a blood‐based biomarker for AD (most often detected as Aβ42/40 ratio) and is associated with neurodegeneration and synaptic loss leading to disease progression.^168^, ^169^ Vais et al. developed a biosensor based on a PPy MIP on screen‐printed carbon electrodes (MIPPy‐SPCE) to perform cyclic voltammetry. The biosensor demonstrated a LoD of 1.2 pg/mL, with successful detection in artificial CSF. Its performance, though, should be assessed in patient‐derived samples in the presence of potential interferents.^92^ Selectivity is indeed challenging in this case. Pereira et al., for example, developed a pioneering biosensor combining a bottom‐up design approach using paper as a platform for the electrochemical recognition of Aβ‐42. The sensor layer produced by the authors relied on a MIP created on the carbon ink electrode's surface by electropolymerising a mixture of the target analyte (Aβ‐42) and the monomer *O*‐phenylenediamine. The linear response of the biosensor reached down to 0.1 ng/mL, and overall, the developed biosensor offered numerous benefits, such as simplicity, low cost, and fast response. Nonetheless, the selectivity and repeatability could benefit from improvement.^99^

**Figure 3:**
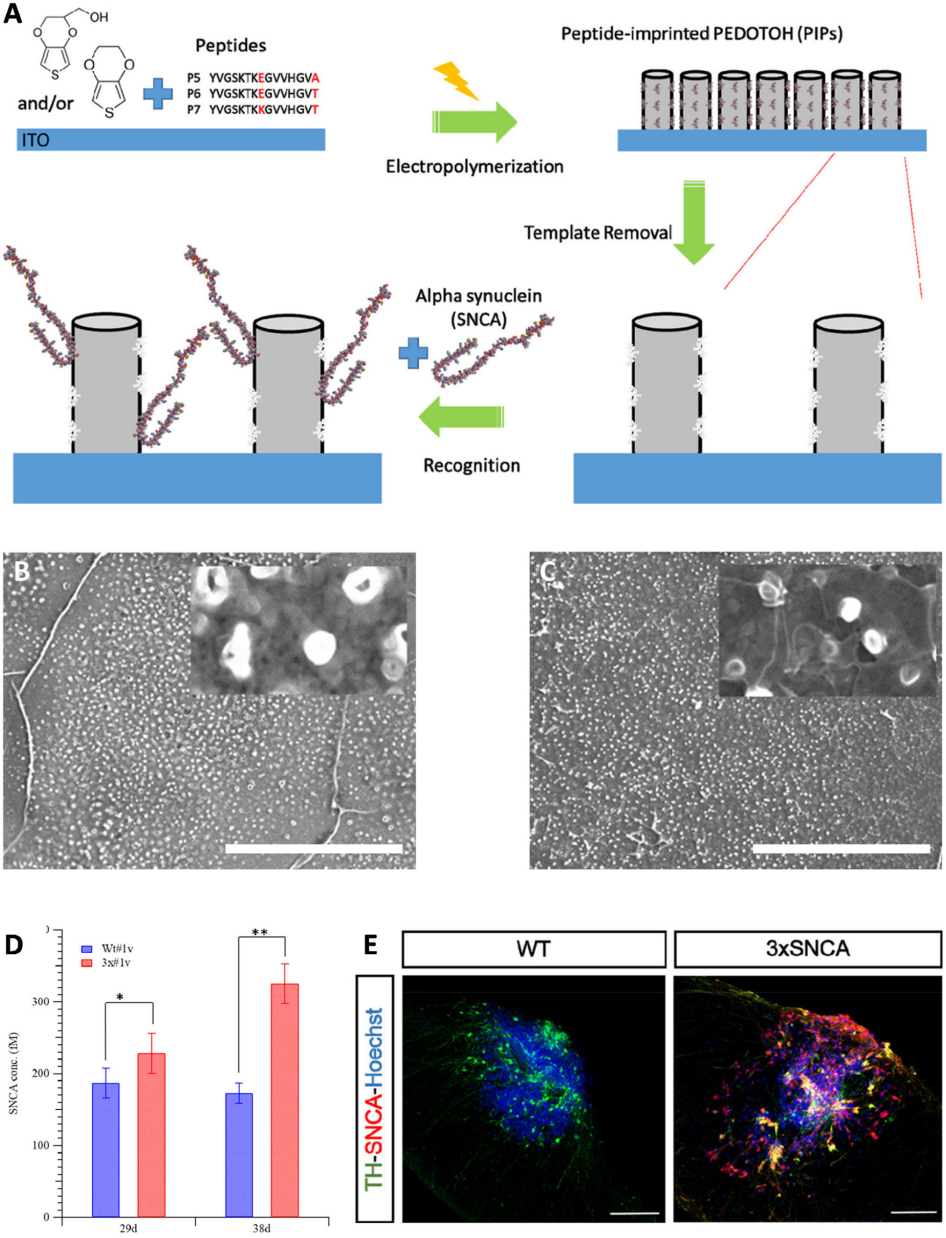
(A) Scheme of the preparation of peptide‐imprinted poly(hydroxy 3,4‐ethylenedioxythiophene)‐coated electrodes and their recognition of α‐synuclein (SNCA). (B, C). Scanning electron microscopy (SEM) images of SNCA peptide imprinted polymers (B) after extracting (template removed), and (C) after rebinding with 0.65 nM (1.0 ng/mL) template molecules, respectively. Scale bars are 50 µm. (D). Comparison of SNCA levels in the media of the healthy individual and the 3× SNCA organoids at Days 29 and 38 (*p < 0.05, **p < 0.001 with respect to the measurement of Wt#1 v at Day 29). (E). Representative maximum intensity projection of confocal images of a WT and 3× SNCA organoids after 30 days of cultivation showing tyrosine hydroxylase (TH, green), SNCA (red), and nucleus (Hoechst, blue). Scale bars are 200 μm. Adapted with permission from Lee et al.^91^, ^98^

In PD, α‐synuclein (SNCA) is well known to disease‐hallmark; high levels of SNCA lead to aberrant aggregation and neurotoxicity. Lee et al. developed a MIP electrochemical sensor for SNCA using the synthetic self‐assembly of poly(hydroxymethyl 3,4‐ethylenedioxythiophene) nanotubes on sensing electrodes. The concentration range of the SNCA peptide sensing was 0.065 pM–0.65 nM, with a LoD as low as 4.0 pM, and SNCA could also be detected in human PD patient‐specific midbrain organoids (Figure 3).^91^, ^98^ Nonetheless, the selectivity and imprinting effect results suggest that there is still significant room for improvement of this sensor.

Dhinesh Kumar et al., instead, focused their efforts on a different PD diagnostic biomarker, the PARK7/DJ‐1 protein. They developed a MIPPy‐SPCE for DJ‐1 protein recognition, with an analytical performance comparable to the corresponding immunosensor in terms of LoD and analytical range, although the performance was only tested in vitro and not on real samples.^94^

Recently, Ayankojo et al. fabricated a sensor for the rapid detection of brain‐derived neurotrophic factor (BDNF), a member of the neurotrophin family of NF proteins associated with the survival and differentiation of neuronal populations, as a potential neurodegenerative disorder biomarker.^170^ An abnormal serum level of BDNF has been linked to the development of AD, schizophrenia and major depressive disorders.^107^, ^171^ With an impressive LoD of 9 pg/mL for BDNF, showing over fivefold selectivity against closely related NF proteins and 30‐fold selectivity against proteins with similar isoelectric point (pI) values (e.g., CD48), BDNF detection was also validated in complex biological samples, highlighting the sensor's potential for real sample analysis.^107^

Sensors were also produced for a variety of other neural targets, including cystine‐bridged cyclic peptide hormones (CBCPHs) [such as oxytocin, atrial natriuretic peptides, and BNPs], which play crucial roles in neural signaling and neuroendocrine regulation. Lin et al. used a custom‐made amphiphilic monomer (Acr‐His‐NHNH‐Fmoc) to form MIPs for CBCPH detection on QCM chips. The produced sensors exhibited pM detection levels, with high selectivity, potentially paving the way for using this monomer to develop other sensors for peptides bearing similar structural moieties.^102^

Of extreme relevance from a neurodiagnostic perspective is the detection of *Neisseria meningitidis*, a human‐specific bacterial pathogen which causes bacterial meningitis by invading the meninges (outer lining) of the central nervous system. Gupta et al. used a computational approach to identify epitopes from outer membrane proteins of *N. meningitidis* and develop MIP QCM sensors. The sensors were able to selectively bind *N. meningitidis* proteins present in the blood serum of patients suffering from brain fever. Therefore, these fabricated sensors could be used as a diagnostic tool for meningitis disease, with a LoD of 1.39 ng/mL and an imprinting factor (expressed as the ratio of the MIP sensor response over the NIP sensor response) >12.^104^, ^105^

Although considerable progress has been made in developing MIP‐based sensors for neurotransmitters and biomarkers, several limitations remain unresolved. A recurring issue is the difficulty in distinguishing structurally similar analytes within complex physiological matrices, such as DA versus catechol or UA versus AA.^172^ Many reported platforms demonstrate high selectivity in idealised systems but lack comparative data in patient‐derived fluids or under interfering ionic backgrounds. Moreover, while innovations such as internal referencing and microelectrode arrays improve performance, the practical integration of these features into scalable, user‐friendly formats for continuous or wearable sensing is still lacking. Bridging the gap between laboratory prototypes and clinically deployable tools will require a shift from single‐analyte optimisation to multiplexed, adaptive platforms tested under real‐world sampling and storage conditions.

#### 4.2.2 Preconcentration and purification of analytes

**Figure 4:**
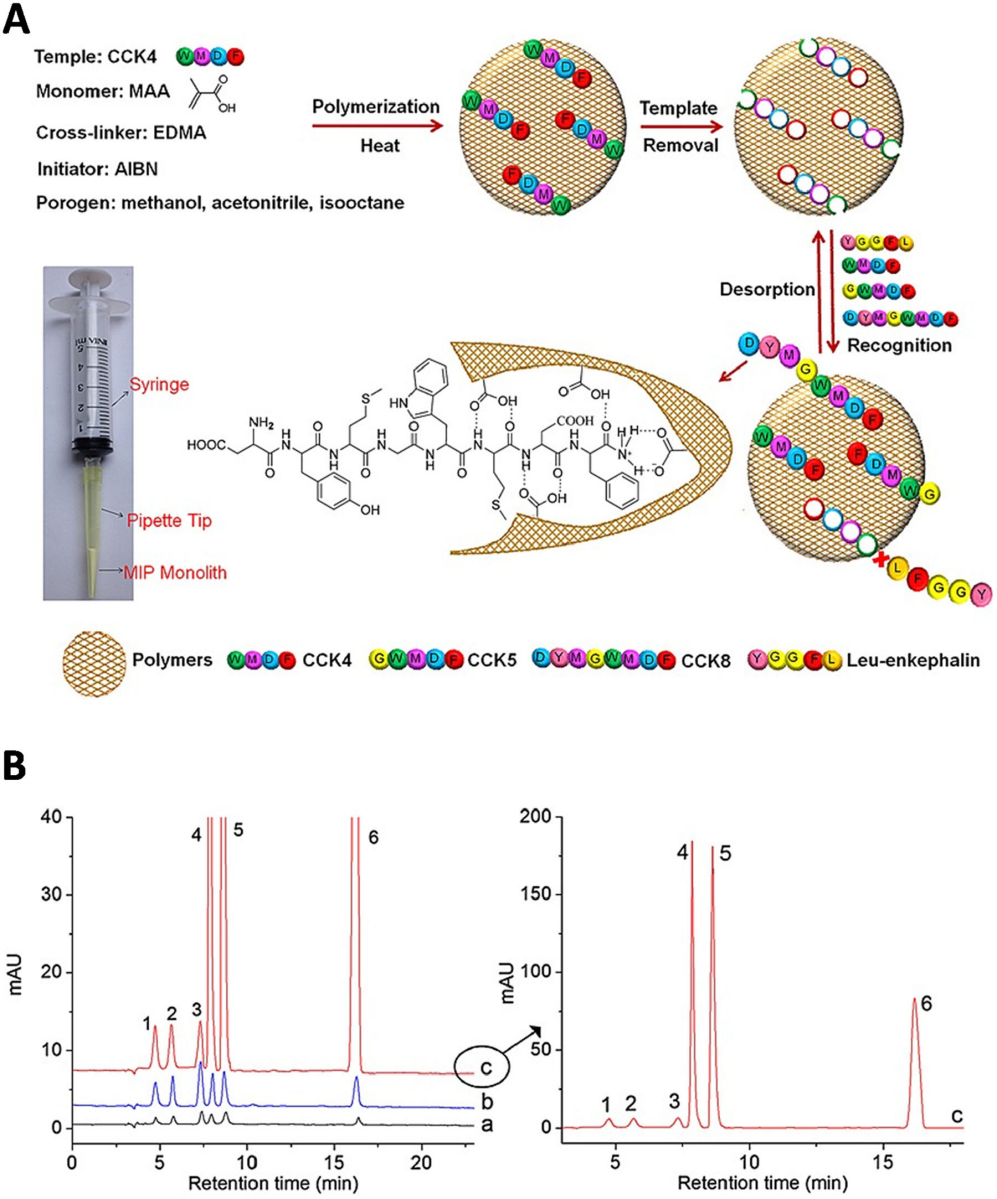
(A) Schematic illustration of the MIP monolith preparation into the pipette tip and recognition procedure for CCK neuropeptides by epitope imprinting technique. (B) Chromatograms of six peptides standard samples obtained by direct HPLC analysis (chromatogram a) and HPLC performed after SPE on NIP (chromatogram b) and MIP (chromatogram c). Peaks: 1, YPLG; 2, Met‐enkephalin (M‐Enk); 3, Leu‐enkephalin (L‐Enk);(l 4, CCK4; 5, CCK5; 6, CCK8. AIBN, 2,2′‐azobisisobutyronitrile; CCK, cholecystokinin; EDMA, ethyleneglyol dimethacrylate; MAA, methacrylate. Adapted with permission from Ji et al.^55^

An important area of investigation is preconcentration and purification of analytes from complex samples as these processes are crucial for achieving high analytical sensitivity, and MIPs show considerable potential in this regard. This is normally done using microparticles or magnetic moieties.^173^ As an example, Ji et al. developed a MIP monolithic column fabricated in a micropipette tip, which could be connected to a syringe to conveniently perform microscale MIP‐SPE to enrich cholecystokinin (CCK) neuropeptides from human CSF before matrix‐assisted laser desorption/ionization time‐of‐flight mass spectrometry (MALDI‐TOF MS) analysis (Figure 4), and proved to be an excellent candidate for in‐pipette‐tip enrichment of multiple endogenous CCK neuropeptides in biofluids simultaneously with high selectivity, sensitivity and excellent reusability.^55^

A similar approach was applied by Claude et al. to improve the sensitivity of CE analysis of neurotransmitters using MIP‐SPE, specifically for DA, 3‐methoxytyramine (3‐MT) and 5‐HT.^174^ The authors had already managed to optimise analysis conditions in buffer, however in urine the salts would reduce the sensitivity. With MIP‐SPE pretreatment, optimal LoDs could be reached (46 nmol/L for DA, 11 nmol/L for 3‐MT and 6 nmol/L for 5‐HT). Huang et al. instead directly generated a MIP monolithic column to perform enantiomeric resolution of different neurotransmitters via CE. Although they used a single template [(‐)‐noradrenaline], the capillary could resolve several racemic neurotransmitters.^53^ It is to be noted, however, that no NIP was tested, and since some cross‐reactivity was observed, a deeper investigation on the imprinting effect should be carried out.

Yuan et al.^93^ attempted a broader application by using novel magnetic graphene oxide (GO)‐MIPs by adopting isoprenaline as dummy‐template to create 3D recognition cavities matching functional groups of catecholamine neurotransmitters. The results showed that MIPs possessed high adsorption capacity, rapid binding rate and excellent selectivity for catecholamine neurotransmitters. On this basis, they were further applied as adsorbents during magnetic SPE for selective recognition and separation of catecholamines (DA, adrenaline, noradrenaline) followed by UPLC‐MS/MS detection. Under the optimum conditions, satisfactory linearity (*r* > 0.99) was obtained with LoDs ranging from 0.53 to 1.93 ng/mL with good reproducibility.

Increased levels of free 3‐nitro‐l‐tyrosine (3NT) have been detected in neurodegenerative tissues,^175^ and also in biological fluids of patients with neurological disorders such as PD.^176^ Moreover, recent data suggest a direct neurotoxic role of this modified amino acid that can cause a selective loss of dopaminergic neurons.^176^, ^177^ To facilitate the analysis of this compound, Mergola et al. developed a MIP‐SPE product and protocol towards 3NT. This product allowed to significantly improve the quality of the samples for analysis, with over 95% 3NT recovery after MIP‐SPE.^46^

Tryptamine belongs to the group of trace bioamines, and is involved in various cardiovascular pathologies such as hypertension, myocardial infarction, and neurovascular pathologies like migraine. The vasoconstriction is related to the trace amine‐associated receptors as well as to 5‐HT receptors due to structural analogy to 5‐HT.^178^, ^179^ Concerning the analysis of tryptamine, the main disadvantages are its low levels (in physiological conditions, its levels are a thousand‐fold lower than those for 5‐HT) and quick conversion into indole‐3‐acetic acid (a metabolite which exhibits even lower concentrations, thus hindering its use for indirect quantification of tryptamine).^180^ Lulinski et al. therefore focused their efforts on the direct preconcentration of tryptamine from CSF, developing a MIP with high affinity and selectivity even in the presence of 5‐HT and TRP in a complex matrix, with tryptamine recoveries ranging between 98.7% and 107.0%.^50^

SPE strategies based on MIPs have enabled efficient enrichment of trace neurochemicals, yet several methodological aspects warrant further consideration. Many systems focus on high recovery for individual targets but offer limited insight into binding specificity when structurally related compounds coexist,^181^ especially in disease‐altered matrices such as CSF or blood. Furthermore, while formats such as pipette‐tip columns or magnetic MIPs offer operational convenience, their long‐term reproducibility and loading capacity across biological batches are rarely quantified. For broader adoption in clinical diagnostics or neurochemical research, future developments should prioritise selectivity benchmarking using complex surrogate samples, evaluate material fatigue over repeated use cycles, and explore automation‐compatible formats for integration into routine workflows.

#### 4.2.3 Neurotransmitters for MIPs production

Although most of the MIP‐based work refers DA used as a template, a number of examples report it used as a functional monomer, since DA is naturally used by mussels to produce an extremely robust polymer at weak alkaline pH values. The first example of this was reported by Zhou et al., who prepared a QCM sensor for the neurotoxin domoic acid, exhibiting a remarkable imprinting effect and selectivity.^182^ The same authors later developed magnetic core‐shell MIP NPs for the imprinting of proteins (human haemoglobin as model template) for purification and/or detection,^183^ and later on another group developed MIP monoliths based on the same polymer which have been successfully used for protein chromatography applications, even in the presence of complex interferents.^54^ The group of Chen et al. has recently reported a series of seminal works on the development of polydopamine (PDA)‐based MIP sensors for protein and amino acids (even specific enantiomers) via Surface Enhanced Raman Spectroscopy (SERS).^184^, ^185^, ^186^ An extremely relevant application for the diagnosis of glioblastoma has been developed by Hashemi‐Moghaddam et al., who used DA to prepare microparticles imprinted with microRNA 21 for the purification and subsequent qPCR analysis of bioptic lysates, dramatically overperforming established extraction methods such as Trizol®.^133^

The utilisation of neurotransmitters like DA as both templates and functional monomers opened up novel directions for MIP chemistry. However, this dual role also introduces ambiguities in mechanistic interpretation. For example, the redox activity and polymerisation reactivity of DA under physiological conditions can complicate imprinting fidelity and reproducibility.^187^ In some cases, polymer networks formed via oxidative self‐assembly may exhibit nonspecific adsorption, thereby masking genuine molecular recognition. Additionally, studies rarely quantify the contribution of secondary interactions, such as hydrophobic stacking or charge repulsion, to binding performance. Addressing these knowledge gaps will require deeper exploration of imprinting dynamics through spectroscopic monitoring, competitive binding assays, and data‐driven modelling.

The representative studies discussed in this section are summarised in Table 2.

**Table 2:** Representative MIP‐based systems for neurotransmitters, and biomarkers.

Application type	Target molecule	Advantages	Disadvantages	Study (reference)
Electrochemical MWNT–MIP sensor	DA	High selectivity for DA over ascorbic acid (AA) and epinephrine (EP); wide linear range (5.0 × 10⁻⁷–2.0 × 10⁻⁴ M); rapid response	Requires acid pretreatment of MWNTs; template removal relies on organic solvents	[148]
Hybrid catalytic electrochemical MIP sensor	Catechol and DA	Specific recognition; wide linear range (228 nM–144 µM); LoD 228 nM; rapid response (~1 s); tolerance to common interferents (AA, serotonin, phenol, etc.); catalytic activity consistent with Michaelis–Menten kinetics	Polymer/initiator synthesis complexity; requires Cu²⁺ preloading; catalytic mechanism not strictly enzyme‐equivalent; long‐term stability only partly assessed	[81]
Electrochemical nanoporous microrod MIP sensor	DA	Au–Ag dealloyed microrod electrode; ultralow LoD (7.63 × 10⁻¹⁴ M); wide linear range (2 × 10⁻¹³–2 × 10⁻⁸ M); high selectivity versus AA, uric acid (UA), DA, phenylethylamine (PEA); validated in serum and brain samples	Requires optimisation of pH and monomer/template ratio; nonconductive polymer layer may affect long‐term stability	[89]
Reversible electrochemical MIP sensor	DA	Fully reversible operation; LoD 760 pM; linear range 0.5–10 nM; reusable ≥30 cycles; successfully detects DA release from PC12 cells	Device fabrication and electrode preparation are complex	[65]
Electrochemical MIP biosensor	Amyloid‐β (Aβ1–42) peptide	LoD 1.2 pg/mL; successful detection in artificial CSF; promising for Alzheimer's disease (AD) biomarker monitoring	Validation with clinical CSF/patient samples lacking; selectivity and long‐term stability require further study	[92]
MIP‐SPE monolithic tip column	Cholecystokinin (CCK4, CCK5, CCK8) neuropeptides	Convenient pipette‐tip format; highly selective enrichment from CSF; reusable; enrichment factors up to 50–60 fold; enabled quantification of endogenous CCK4 (~1 nM)	Limited to laboratory validation; clinical applicability yet to be demonstrated; method requires pH/solvent optimisation	[55]
Magnetic GO‐MIP SPE	Catecholamines (DA, adrenaline, noradrenaline)	Fe₃O₄/graphene oxide (GO) dummy‐template MIPs; high adsorption capacity; rapid binding; excellent selectivity; validated in rat brain tissues; coupled with UPLC‐MS/MS	Limited to laboratory validation; further optimisation required for clinical application	[93]
MIP‐SPE cartridge	3‐nitro‐l‐tyrosine (3NT)	Highly selective water‐compatible MIP; >90–95% recovery from spiked urine; LOD 0.7 µg/mL; improved sample clean‐up for neurodegenerative marker analysis	Validation limited; tested only on spiked samples, no patient cohort yet	[46]
QCM sensor	Domoic acid (dummy template: 1,3,5‐benzenetricarboxylic acid)	Strong imprinting effect; polydopamine‐based film with high selectivity and affinity; LoQ 5 ppb; validated in mussel extracts	Validation scope limited; only tested in mussel samples, no broader/clinical validation	[182]

Abbreviations: 3NT, 3‐nitro‐l‐tyrosine; AA, ascorbic acid; Aβ_1–42_, amyloid‐beta (1–42) peptide; ACCK4, cholecystokinin tetrapeptide; AD, Alzheimer's disease; Au–Ag, gold–silver; CCK5, cholecystokinin pentapeptide; CCK8, cholecystokinin octapeptide; CSF, cerebrospinal fluid; Cu²⁺, copper(II) ion; DA, dopamine; EP, epinephrine; Fe₃O₄, magnetite [iron(II, III) oxide]; GO, graphene oxide; LoD, limit of detection; LoQ, limit of quantification; MIP, molecularly imprinted polymer; MWNT, multi‐walled carbon nanotube; PC12, pheochromocytoma (rat adrenal medulla–derived) cell line; PEA, phenylethylamine; ppb, parts per billion; UA, uric acid; UPLC‐MS/MS, ultra‐performance liquid chromatography–tandem mass spectrometry.

### 4.3 MIPs for therapy: Drug development, drug delivery and direct bioactivity

Important areas where MIPs have been developed for neurological applications are drug development and drug delivery. In certain instances, direct bioactivity of MIPs was also reported, aimed at addressing a variety of neurological conditions.^188^

For example, MIPs have been developed to explore compounds with neuroprotective capacity from natural sources and herbs as well as concentrating them for analysis within bodily fluids and tissues (e.g., Pichroside II, triptolide, Schisandrol A).^117^, ^118^, ^120^ A quite interesting application has been developed by Naklua et al., who described MIP microprobes to assess small molecules interacting with a neural sensing protein in the hypothalamus. The authors elucidated subtle processes of therapeutic relevance, by controlling the temperature responsible for monitoring the neuronal activity and its neuroplasticity.^48^ Although the authors claimed that this approach could accelerate the discovery of the neuroleptic potential of drugs or new drug candidates, it might be challenging to extend it to non‐hypothalamic regions of the brain parenchyma.

Other applications were reported for the analysis of the pharmacokinetics of neuroleptic drugs (e.g., haloperidol, tramadol, Schisandrol A) in different tissues, exploiting MIPs to extract the drugs from various complex matrices (e.g., brain tissue),^47^, ^49^, ^118^ although some sample loss was observed depending on the type of drug imprinted.

In another instance, a MIP sensor was developed for the therapeutic dosage monitoring of methamphetamines in urine and blood, exploiting a new nanocomposite between MIPs and carbon quantum dots.^127^ A similar approach was also used to develop a sensor to monitor the dosage of the antidepressant citalopram and DA.^129^, ^136^ Nonetheless, some performance loss was observed after multiple uses, although the production method was sustainable and environmental friendly.

**Figure 5:**
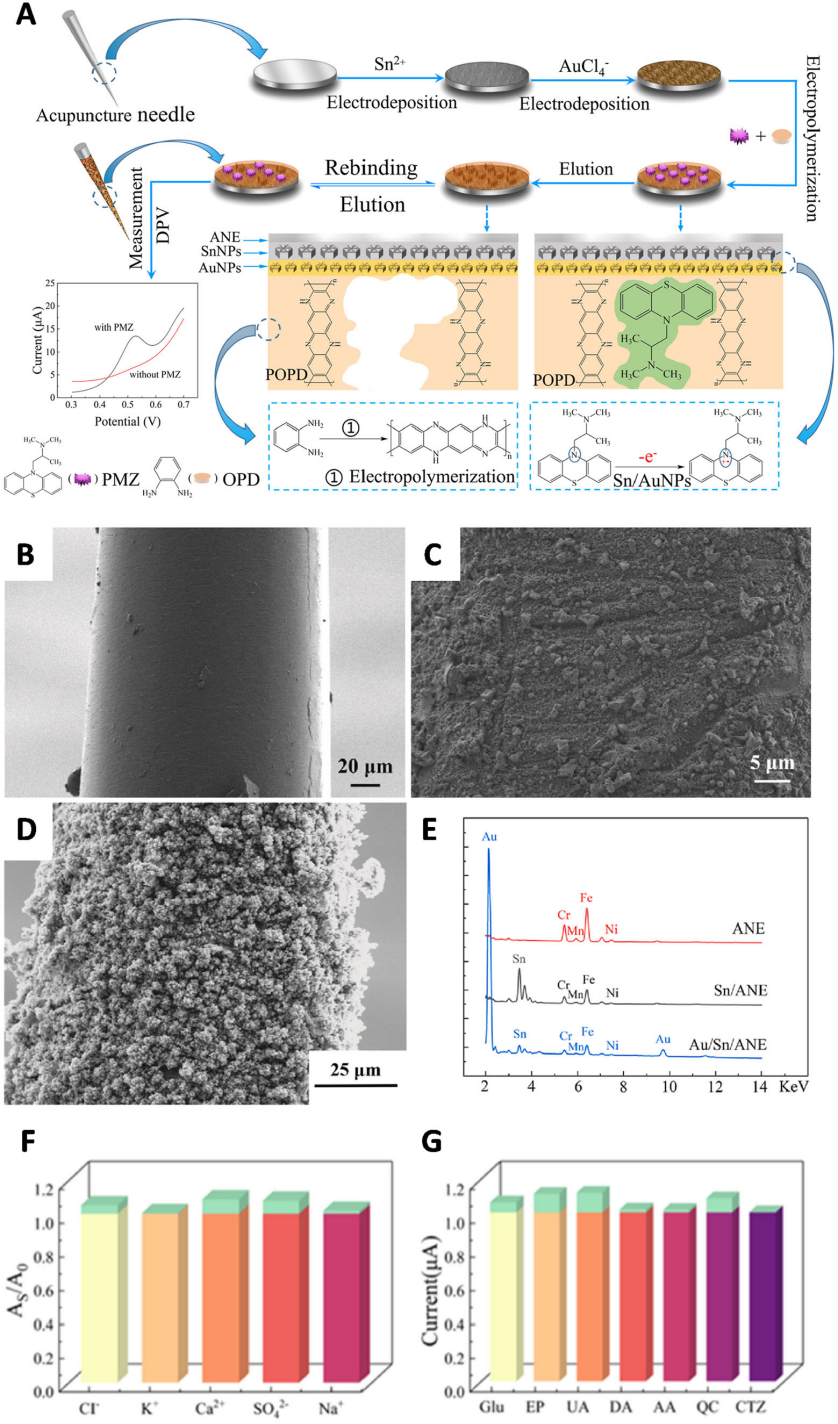
(A) Scheme of the construction of MIP needle sensor for electrochemical evaluation of promethazine (PMZ). (B–E). SEM images of bare acupuncture needle electrode (B), after Sn electrodeposition (C), and after Au electrodeposition (D), with the corresponding energy‐dispersive spectroscopy (EDS) spectra (E). (F). Current responses of the MIP sensor (during differential pulsed voltammetry) to phosphate buffer solution (PBS) containing 0.1 mM PMZ with 1 mM interfering ions (Cl^‐^, K^+^, Ca^2+^, SO_4_^2−^, or Na^+^. (G). Current responses of the MIP sensor (during differential pulsed voltammetry) to PBS containing 0.1 mM PMZ with 0.1 mM glucose (Glu), epinephrine (EP), uric acid (UA), dopamine (DA), ascorbic acid (AA), quercetin (QC) and chlorothiazide (CTZ). ANE, acupuncture needle electrode; AuNPs, Au nanoparticles; DPV, differential pulse voltammetry; OPD, o‐phenylenediamine; SnNPs, Sn nanoparticles. Adapted with permission from Liu et al.^100^

Liu et al. developed an extremely useful electrochemical MIP sensor shaped as an acupuncture needle for the detection and quantification of promethazine, a widely used therapeutic agent and nerve tranquiliser for mental disorders. It is critical to monitor this drugs' dosage in patients and in the environment as its excessive use may cause serious side effects, such as pseudo‐PD and potential respiratory depression. The sensor was produced first via the electrodeposition of Sn/AuNPs, followed by the electropolymerisation of *o*‐phenylethilendiamine, and it exhibited a selective recognition capacity and good reproducibility even in the presence of interferents in high concentration (Figure 5).^100^

Along these lines, Choudhary et al. developed MIP‐carbon paste electrodes for the monitoring of antiepileptic drugs phenobarbital, carbamazepine, and levetiracetam, which are characterised by a narrow therapeutic window requiring adequate dosage regulation.^122^ The developed sensors allowed to obtain a response within 2 min from complex matrices such as whole blood or plasma. Compared with existing tests, these sensors offer a faster and more accurate way to monitor these antiepileptic drugs, which is crucial for optimising therapy and improving patient outcomes. Nonetheless, further optimisation is required to avoid saturation phenomena due to the presence of aqueous media.

An extremely relevant application has been reported by Naklua et al., who developed a MIPs against the neurotransmitters DA and 5‐HT not for analytical purposes, but to guide the reaction of a library of synthetic chemical fragments, with the aim of identifying successful receptor agonists (potentially for the treatment of PD). The screening test of the identified compounds with natural D1 receptor yielded *K*_d_ values of 100–500 nM. Thus, this strategy allowed to achieve a promising preclinical testing to identify possible neuroleptic agents and their neuroprotective effects, hopefully with reduced side effects.^67^

Drug delivery strategies play an important role in the treatment of neurological diseases, for example, brain tumours (e.g., glioma). Wang et al. were amongst the first to develop and test a chitosan‐alginate LbL MIP microcapsule for sustained doxorubicin release towards glioma treatment.^114^ Although the release of the drug was sustained for almost 1 week, the difference between MIP and NIP both in terms of drug release and cytotoxic effect on glioma cells required optimisation. One fundamental limitation in this context, however, lies in the incomplete understanding of the release mechanism. It remains unclear whether the observed sustained release results from specific template–polymer interactions within the imprinted cavities or from nonspecific diffusion through the polymer network.^189^ This distinction is crucial for rational polymer design, as only the former mechanism ensures responsive and selective release across different tissue environments. Future studies should systematically decouple these effects (e.g., through kinetic modelling and carefully designed control experiments). To adjuvate the postsurgical chemotherapy, Bărăian et al. developed a fibrin‐based hydrogel containing a MIP NPs‐based drug reservoir to be injected within the tumour post‐resection cavity for the localised and sustained release of ruxolitinib.^112^

Another group exploited an epitope of the nicotinic acetylcholine receptor α7 (nAchR α7) as a template to develop MIP NPs capable of targeting the brain (e.g., for delivery purposes). The system provided promising results both in vitro and in vivo.^190^ It needs to be noted, however, that the MIP NPs were based on a nonbiodegradable methacrylate polymer, which could hinder their translational potential.

An extremely elegant delivery system aimed at both biodegradability and specific brain targeting was developed by Asadi et al. to selectively release olanzapine (an antipsychotic used to treat schizophrenia) into the brain by exploiting a fructose‐based monomer and cross‐linker.^132^ By cleverly taking advantage of the fructose brain metabolism as a targeting and degradation system, the authors managed to significantly improve the biodistribution of olanzapine by increasing its half‐life and reducing hepatic first passage. Nonetheless, the complexity of the synthesis of the nanocarrier and of the fructose monomer/cross‐linker could hinder the translation of this product to the clinic due to increased overall costs. Beyond synthetic complexity, the biological variability associated with metabolism‐based targeting strategies also requires closer scrutiny. Since the targeting mechanism relies on endogenous fructose metabolism, interindividual differences in enzyme levels or metabolic rates may lead to inconsistent biodistribution outcomes.^191^ Moreover, many sugar‐derived monomers are chemically labile and susceptible to batch‐to‐batch variability, posing additional manufacturing challenges for clinical translation. Addressing these variables through rigorous standardisation and in vivo validation will be essential to fully realise the potential of such systems.

Similarly, by exploiting the presence of ApoE receptors in the BBB, Figueiredo et al. developed and evaluated MIP NPs conjugated with ApoE as a possible drug nanocarrier to the brain with a model drug (donepezil).^147^ Although the NPs targeted with ApoE exhibited a twofold increased permeability on a BBB model, they exhibited some cytotoxicity and haemolytic activity, suggesting that further optimisation is required to increase the biocompatibility of the envisaged nanosystems.

Trotta et al. attempted to solve the l‐DOPA degradation issues for PD treatment by developing MIP cyclodextrin‐based nanosponges (MIP‐NSs) for the prolonged and controlled release of l‐DOPA. The MIP‐NSs successfully exhibited a slower and more prolonged release profile than the NIP‐NSs (over days instead of hours), without any observed degradation of the l‐DOPA cargo hosted in the MIP‐NSs.^66^

Pursuing a timely 3Rs (Replacement, Reduction and Refinement of animals in research) angle, since 2016, researchers. have been extensively investigating the use of MIPs and MIP NPs to perform the high‐throughput screening and isolation of compounds capable of interrupting the interaction of neuronal nitric oxide synthase (nNOS) and post‐synaptic density protein‐95 (PSD‐95) with the aim of evaluating potential drug candidates. Animal or cell experiments would normally be used to evaluate these candidates, but these experiments are complex, expensive, and time‐consuming (usually 2 days per compound) because they are subject to complicated model construction, reperfusion, and co‐immunoprecipitation. With this approach, the author managed to significantly reduce the timings and resources required to isolate and identify new drug candidates to treat stroke damage, and confirmed their success with both in vitro and in vivo testing of the isolated candidates.^115^, ^116^, ^134^, ^143^, ^192^, ^193^

In terms of direct bioactivity, MIPs have been developed to try and address the accumulation of amyloid‐beta protein in the brain which correlates with the cognitive impairment observed in neurodegenerative pathologies such as AD. The authors developed a MIP membrane towards the amyloid‐beta protein fragment 25‐35, the more neurotoxic domain of amyloid‐beta protein, with the aim to ultimately remove it from the blood and the CSF. Although the preliminary results were quite promising in terms of rebinding performance, porosity and mechanical properties, no testing was performed on the actual blood or CSF, which would be paramount to translate this technology to the clinic.^71^ Another group attempted a slightly different approach aimed at inhibiting the amyloid‐beta protein aggregation instead of promoting the clearance. As a model target, they used lysozyme (since some natural variants of human lysozyme are associated with systemic non‐neurological amyloidosis that leads to amyloid protein fibril deposition). The authors prepared biocompatible targeted, highly loaded curcumin lysozyme‐MIP NPs that could effectively and specifically inhibit the aggregation of amyloid fibrils in a dose‐dependent manner and significantly inhibit the structural transition of lysozyme from an α‐helix to a β‐sheet conformation. It would be interesting, though, to observe the feasibility of this approach towards other types of amyloid precursors or amyloid fibres, especially in vivo.^142^ In addition, the precise mechanism by which these MIPs suppress protein aggregation remains to be fully elucidated. In some cases, inhibition of fibril formation could stem from nonspecific surface adsorption or steric hindrance rather than from selective template recognition. This distinction is especially important when targeting dynamic aggregation‐prone conformations, where specificity may determine therapeutic efficacy.^194^ Comparative studies using NIP or other MIP controls and quantitative assessments of intermediate binding would help clarify whether the observed inhibition is truly MIP‐driven.^194^, ^195^

In a related work, Lee et al. attempted to address the aggregation of SNCA, which is observed in PD and other conditions, by imprinting a peptide from SNCA onto magnetic MIP NPs both for diagnostic and potential therapeutic purposes.^135^ Although the diagnostic potential was clear, the therapeutic envisaged in vivo translation would still need optimisation. Particularly, it would need to be confirmed that the produced MIP NPs can cross the BBB to exert their effect without causing brain or other organ damage at the required dosage.^128^

Another direct action is related to the potential to develop nano‐imprinted structured materials and MIPs to guide nerve regeneration, develop BBB models or aid with neurointerfacing towards neuromodulation.^72^, ^73^, ^188^, ^196^, ^197^ A successful example has been reported by Fallah‐Darrehchi et al., who developed an electrospun poly(l‐lactide‐*co*‐d,l‐lactide) (PLDLLA) mat containing MWNTs and 4‐aminopyridine (4‐AP)‐loaded MIP NPs (MIP4‐AP). In this way, they obtained an electrically conductive matrix loaded with a controlled release system for a drug aimed at improving impulse conduction.^130^ Their results were extremely promising, as the in vitro assessment of PC‐12 neuroblastoma cells on optimal PLDLLA/MWNTs/MIP4‐AP nanofibrous samples revealed the highest cell proliferation without cytotoxicity compared to neat nanofibers, whilst in vivo, the same system was able to repair the rat's sciatic nerve after 4 weeks (in accordance with sciatic function index and histological studies). It would be interesting to assess the long‐term performance of this regeneration system, particularly in terms of biodegradation and elimination.

For ease of consultation and clarity, the studies discussed in this section are summarised in Table 3.

**Table 3:** Representative MIP‐based systems for neurological diseases and therapies.

Application type	Target Molecule	Advantages	Disadvantages	Study (reference)
UHPLC–MS/MS pharmacokinetic profiling	Triptolide (rat brain, blood microdialysates)	High sensitivity (LoD 0.45–0.50 pg/mL; LoQ 3.0 pg/mL); wide linear range (*R*² >0.995); acceptable intra‐/inter‐day precision and accuracy; >96% derivatisation efficiency; no significant matrix effect; applicable to in vivo PK studies in rats (brain and blood)	Requires isotope‐label derivatisation, increasing complexity and cost; validated only in rat samples	[117]
Electrochemical microneedle MIP sensor	Promethazine	Good linearity (0.5–500 µM, *R*² high); low detection limit (LoD 0.14 µM, S/N = 3); high repeatability, stability and selectivity; suitable for human serum analysis	Response varies with pH; may limit use in environmental monitoring	[100]
Disposable electrochemical MIP sensor	Phenobarbital, Carbamazepine, Levetiracetam	Rapid measurement (<2 min); linear range 0–60 μg/mL for phenobarbital and levetiracetam, 0–12 μg/mL for carbamazepine, covering respective therapeutic ranges; negligible interference from whole blood and plasma; suitable for point‐of‐care monitoring of antiepileptic drugs	Risk of signal saturation in aqueous media; broader clinical validation is still required	[122]
MIP‐based receptor screening assay	DA, 5‐HT	Enabled fragment‐based screening of dopamine/serotonin analogues; DS‐MIP showed higher binding capacity (Bmax ~1.0 M/g, ~2× d‐MIP); good binding affinity to 5‐HT1B receptors (Kd 100–500 nM); potential to identify new D3 receptor agonists for Parkinson's disease with reduced side effects	Applicability beyond selected receptor subtypes not yet demonstrated; findings mainly from docking and preclinical assays, requiring further in vivo validation	[67]
MOF‐based MIP solid‐phase extraction and bioactivity assay	nNOS–PSD‐95 uncouplers (from Sanhuang Xiexin Decoction)	High binding capacity, fast kinetics, and excellent selectivity; effective for solid‐phase extraction with HPLC; demonstrated neuroprotective and anti‐stroke activity in vitro and in vivo	Requires further validation to confirm clinical translatability and reproducibility across biological systems	[143]
MIP microcapsule drug delivery system	Doxorubicin	High loading capacity (up to 155.1 μmol g⁻¹); sustained release >168 h; good biocompatibility; enhanced glioma inhibition versus free drug	Release triggered by pH changes; mechanism of release (specific vs. nonspecific) remains unclear; validation limited to in vitro and glioma cell model	[114]
Biodegradable fructose‐based MIP nanocarrier for brain drug delivery	Olanzapine	Biodegradable and biocompatible carrier; magnetic guidance enhances brain targeting; fructose metabolism improves biodistribution and half‐life; validated in vitro and in vivo	Complex synthesis and formulation; variability due to metabolism‐based targeting; long‐term clinical applicability yet to be confirmed	[132]
Drug delivery (Parkinson's therapy)	L‐DOPA	Cyclodextrin‐based MIP nanosponges enable slow and prolonged release; stable encapsulation prevents L‐DOPA degradation; strong ability to sustain release confirmed by in vitro studies	Long‐term safety and biodegradability not fully assessed; requires further validation for oral administration	[66]
Direct bioactivity/protein removal	Amyloid‐β fragment 25–35	Selective membrane binding to the neurotoxic amyloid‐β fragment; proof‐of‐concept for extracorporeal removal from blood and cerebrospinal fluid; biocompatible polymer backbone with improved imprinting via carboxyl modification	No validation in real blood or CSF samples; results limited to in vitro rebinding assays	[71]
Direct bioactivity/protein aggregation inhibition	Lysozyme (amyloid fibril model)	Dose‐dependent inhibition of amyloid fibril aggregation; reduced hydrophobic interactions; good biocompatibility	Mechanism not fully clarified; possible contribution from nonspecific adsorption	[142]
Direct bioactivity/protein aggregation inhibition	α‐Synuclein (SNCA) peptide	Magnetic MIP NPs specifically bind SNCA monomers and fibrils; demonstrated potential for both diagnostic detection and therapeutic clearance in Parkinson's disease models	BBB penetration and in vivo safety are yet to be proven	[135]
Nerve regeneration/controlled drug delivery	4‐aminopyridine (4‐AP)	Conductive PLDLLA/MWCNT scaffold with MIP‐4AP enables sustained release (~96 h), improved electrical conductivity, enhanced PC12 cell proliferation and viability, effective sciatic nerve repair in vivo	Long‐term biodegradation and elimination remain unverified	[130]
Metabolic disorder therapy (Phenylketonuria, PKU)	Phenylalanine (Phe)	Oral Phe‐selective MIPs reduced blood Phe by ~46%–48% in PKU mouse model; restored hippocampal dopamine levels; improved locomotor and cognitive function	Nonbiodegradable methacrylate backbone; potential risk of long‐term accumulation	[52]

Abbreviations: 4‐AP, 4‐aminopyridine; 5‐HT, serotonin; amyloid‐β, amyloid beta peptide; BBB, blood–brain barrier; Bmax, maximum binding capacity; CSF, cerebrospinal fluid; D3, dopamine D3 receptor; DA, dopamine; D‐MIP, dopamine MIP; DS‐MIP, dopamine‐serotonin MIP; Kd, dissociation constant; L‐DOPA, levodopa (L‐3,4‐dihydroxyphenylalanine); LoD, limit of detection; LoQ, limit of quantification; MIP, molecularly imprinted polymer; MOF, metal–organic framework; MWCNT, multi‐walled carbon nanotube; nNOS, neuronal nitric oxide synthase; PC12, pheochromocytoma (rat adrenal medulla–derived) cell line; Phe, phenylalanine; PLDLLA, poly(L,d‐lactide); PK, pharmacokinetic; PKU, phenylketonuria; PSD‐95, postsynaptic density protein 95; S/N, signal‐to‐noise ratio; SNCA, alpha‐synuclein; UHPLC–MS/MS, ultrahigh‐performance liquid chromatography–tandem mass spectrometry.

## 5 CHALLENGES AND FUTURE DIRECTIONS

MIPs hold tremendous promise for advancing the field of neurology by offering highly specific diagnostic and therapeutic tools tailored to the complex and multifaceted challenges of neurological disorders. In recent years, important progress has been made: MIPs have demonstrated remarkable selectivity and stability across diverse templates including neurotransmitters, peptides, and proteins, and their integration with electrochemical and optical platforms has enabled sensitive detection in biofluids such as blood, saliva, and CSF. Their successful application in other biomedical contexts, including bone biomarker monitoring and antiviral therapeutic design,^198^, ^199^ further illustrates the adaptability of MIP technology and strengthens the case for its future impact in neurology. Moreover, their robustness and low‐cost production offer a practical advantage over antibodies and natural receptors, particularly for settings where cold‐chain logistics are impractical.

Despite their potential, though, several significant challenges must be addressed to fully harness their benefits. Ensuring the biocompatibility and safety of MIPs is critical, particularly for applications involving direct interactions with sensitive neural tissues or prolonged systemic exposure.^125^, ^128^ While MIPs offer high selectivity and stability, their direct use in vivo remains limited due to concerns regarding toxicity, immunogenicity, and non‐biodegradability. As previously mentioned, most conventional MIPs are based on methacrylate or acrylamide backbones that are nondegradable and may accumulate in tissues over time.^199^ Strategies to improve biocompatibility and degradability include the incorporation of natural polymers (such as chitosan or gelatin), the use of biodegradable cross‐linkers (e.g., ester or disulfide bonds), and the development of stimuli‐responsive MIPs that degrade under physiological triggers such as pH or enzymatic activity. In addition, surface modification techniques (e.g., PEGylation or zwitterionic coatings) can and have been employed to reduce immune recognition and nonspecific adsorption.^40^ However, thorough evaluation through cytotoxicity assays, immunogenic profiling, and in vivo biodistribution studies remains essential before any clinical translation can be envisaged.^124^

To provide a balanced perspective, it is important to highlight both the advantages and drawbacks of current MIP platforms. Advantages include their robustness under harsh conditions, the ability to be tailored toward a wide range of targets, cost‐effectiveness, and long‐term stability compared to biological recognition elements. Drawbacks, however, include limited biodegradability, reduced performance in complex biological matrices, batch‐to‐batch variability, and the lack of standardised regulatory frameworks. This duality underscores the need for further optimisation, guided by the 3Rs ethical framework^200^ in preclinical research to develop MIPs with enhanced biocompatibility and environmental sustainability,^201^ before routine neurological use can be realised.

The scalability of production processes represents another hurdle, as current synthesis methods may not be economically or logistically viable for widespread clinical implementation.^17^, ^20^ Regulatory approval processes for both diagnostic and therapeutic applications of MIPs remain a bottleneck, requiring robust evidence of safety, efficacy, and reproducibility in preclinical and clinical settings. Furthermore, the regulatory landscape for these materials, particularly for neurological applications, is still emerging, necessitating close collaboration between researchers, industry stakeholders, and regulatory agencies.^124^, ^202^ Although MIPs have shown encouraging results in laboratory settings, their adoption in clinical hospital workflows remains extremely limited. This gap arises not only from regulatory barriers but also from practical incompatibilities with real‐world medical environments. Many reported MIP‐based sensors and delivery systems are designed for idealised conditions, lacking systematic validation in clinically relevant matrices such as whole blood, saliva, or CSF from diverse patient populations. Furthermore, most hospital settings demand diagnostic tools that are fully integrated, rapid, and operable by nonspecialist staff, whereas many MIP systems still require complex sample pretreatment, electrochemical measurement steps, or reconditioning between uses. In the context of drug delivery, concerns related to long‐term accumulation of nonbiodegradable polymers, poorly defined degradation products, or inconsistent release behaviour further complicate translation into regulated clinical use. Bridging these gaps will require not only improved material biocompatibility and batch reproducibility, but also early‐stage codesign with clinicians, bioengineers, and health economists to ensure that MIP‐based solutions can be seamlessly embedded into hospital diagnostic and treatment pathways.

A critical challenge limiting the translational potential of MIPs lies in the discrepancy between in vitro binding affinity and in vivo recognition performance. While MIPs can exhibit high template affinity under controlled laboratory conditions, their selectivity often deteriorates in biological environments due to matrix complexity, competitive adsorption, or conformational changes in target molecules.^203^ This drop in performance is especially pronounced when MIPs encounter proteins or metabolites with a similar size, charge, or hydrophobicity to the intended target. Strategies to enhance binding affinity and maintain recognition fidelity in vivo include the incorporation of hydrophilic or zwitterionic monomers to reduce nonspecific binding, the use of flexible or stimuli‐responsive polymers to adapt to dynamic biological contexts, and surface imprinting techniques that expose recognition sites while preserving accessibility.^204^ Furthermore, selecting imprinting conditions that mimic physiological pH, ionic strength, and temperature can improve the relevance and robustness of the resulting binding cavities.^205^ In parallel, introducing hierarchical porosity or integrating MIPs with adaptive materials like MOFs may enhance analyte diffusion and fine‐tune recognition kinetics.^206^

Looking forward, several future directions can be prioritised. First, material design should focus on creating biodegradable, stimuli‐responsive, and adaptive MIPs capable of dynamic performance in complex neural environments. Second, although most reported MIPs for neurological applications have been synthesised via conventional free radical polymerisation, controlled radical polymerisation techniques such as RAFT and ATRP offer promising opportunities for improved structural control, biocompatibility, and functional performance. These approaches may play a key role in the next generation of MIP‐based systems, particularly for biomedical applications requiring precise molecular architecture and reduced batch variability.^17^ Third, combining MIPs with neural interfaces, implantable electronics, and bioelectronic platforms can enable real‐time monitoring and intervention. Fourth, artificial intelligence (AI)‐driven modelling and machine learning can accelerate template selection, monomer design, and predictive optimisation, reducing trial‐and‐error development.^207^ Finally, early and continuous codesign with clinicians, regulatory experts, and health economists will ensure that translational barriers are identified and mitigated at the outset.

Collaboration between interdisciplinary teams, including neuroscientists, material scientists, chemists, and clinicians, is essential for translating MIPs from the laboratory to clinical practice. Rigorous preclinical and clinical studies are necessary to evaluate their performance under realistic conditions, including complex biological matrices and patient‐derived samples. Moreover, fostering partnerships between academia, industry, and regulatory bodies can streamline the path toward commercialisation and ensure that MIP‐based solutions meet the highest standards of safety and efficacy.^199^, ^202^

In conclusion, although neurology represents an always evolving frontier where innovations in diagnostics and therapeutics are critical for addressing the growing burden of neurological disorders, MIPs offer a cutting‐edge approach to tackling these challenges, thanks to their specificity and versatility. By situating current advances alongside their unresolved drawbacks, and by articulating future opportunities in material design, AI‐driven optimisation, and clinical integration, a clearer translational roadmap emerges. This progress not only promises significant improvements in patient outcomes but also paves the way for a deeper understanding of the brain and its disorders, ultimately enhancing the quality of life for individuals affected by these conditions.

## AUTHOR CONTRIBUTIONS

Alessandro Poma developed the first idea for the study and wrote the first draft of the manuscript. All authors contributed to the study's concept, literature search, and analysis. All authors edited, reviewed, and approved the final draft of the manuscript.

## CONFLICT OF INTEREST STATEMENT

The authors declare no conflicts of interest.

## ETHICS STATEMENT

Not applicable.

## Data Availability

The manuscript does not contain novel data, and all the information provided can be sourced from literature.

## References

[1] Thakur KT , Albanese E , Giannakopoulos P , et al. Neurological disorders. In: Patel V , Chisholm D , Dua T , Laxminarayan R , Medina‐Mora ME , eds. Mental, Neurological, and Substance Use Disorders: Disease Control Priorities. 4, Third Edition. The International Bank for Reconstruction and Development/The World Bank© 2016 International Bank for Reconstruction and Development/The World Bank; 2016. 27227198

[2] Augustine JR . Human Neuroanatomy. Wiley; 2016.

[3] Klöppel S , Abdulkadir A , Jack Jr. CR , Koutsouleris N , Mourão‐Miranda J , Vemuri P . Diagnostic neuroimaging across diseases. Neuroimage. Jun 2012;61(2):457‐463. 10.1016/j.neuroimage.2011.11.002 22094642 PMC3420067

[4] Mortimer AM , Likeman M , Lewis TT . Neuroimaging in dementia: a practical guide. Pract Neurol. 2013;13(2):92‐103. 10.1136/practneurol-2012-000337 23468560

[5] Bhat S , Chokroverty S . Electroencephalography, electromyography, and electro‐oculography: general principles and basic technology. In: Chokroverty S , ed. Sleep Disorders Medicine. Springer New York; 2017:295‐330. Chapter 18.

[6] Gomes HR . Cerebrospinal fluid analysis: current diagnostic methods in central nervous system infectious diseases. Arq Neuropsiquiatr. 2022;80(5 suppl 1):290‐295. 10.1590/0004-282X-ANP-2022-S114 35976304 PMC9491443

[7] Staal SL , Olie SE , van Weeghel M , et al. Cerebrospinal fluid metabolome in central nervous system infections: a study of diagnostic accuracy. Ann Neurol. 2025;98:851‐863. 10.1002/ana.27291 40525505 PMC12542320

[8] Reddy DS , Abeygunaratne HN . Experimental and clinical biomarkers for progressive evaluation of neuropathology and therapeutic interventions for acute and chronic neurological disorders. Int J Mol Sci. 2022;23(19):11734. 10.3390/ijms231911734 36233034 PMC9570151

[9] Grossberg GT . Cholinesterase inhibitors for the treatment of Alzheimer's disease: getting on and staying on. Curr Ther Res. 2003;64(4):216‐235. 10.1016/S0011-393X(03)00059-6 24944370 PMC4052996

[10] Liu W , Shen Y , Zhong Y , et al. Levodopa improved different motor symptoms in patients with Parkinson's disease by reducing the functional connectivity of specific thalamic subregions. CNS Neurosci Ther. 2024;30(2):e14354. 10.1111/cns.14354 37452488 PMC10848087

[11] Bulaj G . Combining non‐pharmacological treatments with pharmacotherapies for neurological disorders: a unique interface of the brain, drug‐device, and intellectual property. Front Neurol. 2014;5:126. 10.3389/fneur.2014.00126 25071711 PMC4095562

[12] Ellenbogen R , Sekhar L , Kitchen N . Principles of Neurological Surgery. Elsevier Health Sciences; 2017.

[13] Costanzi S , Machado JH , Mitchell M . Nerve agents: what they are, how they work, how to counter them. ACS Chem Neurosci. 2018;9(5):873‐885. 10.1021/acschemneuro.8b00148 29664277

[14] Jett DA , Spriggs SM . Translational research on chemical nerve agents. Neurobiol Dis. 2020;133:104335. 10.1016/j.nbd.2018.11.020 30468862

[15] Adampourezare M , Hasanzadeh M , Nikzad B . Recent progress and challenges in the application of molecularly imprinted polymers for early‐stage screening of neurodegenerative diseases‐related protein biomarkers. Microchem J. 2023;192:108931. 10.1016/j.microc.2023.108931

[16] Pilvenyte G , Ratautaite V , Boguzaite R , et al. Molecularly imprinted polymers for the recognition of biomarkers of certain neurodegenerative diseases. J Pharm Biomed Anal. 2023;228:115343. 10.1016/j.jpba.2023.115343 36934618

[17] Poma A , Turner APF , Piletsky SA . Advances in the manufacture of MIP nanoparticles. Trends Biotechnol. 2010;28(12):629‐637. 10.1016/j.tibtech.2010.08.006 20880600

[18] Poma A , Whitcombe M , Piletsky S . Plastic antibodies. In: Piletsky SA , Whitcombe MJ , eds. Designing Receptors for the Next Generation of Biosensors. Springer; 2013:105‐129. chap 105.

[19] Patel KD , Kim HW , Knowles JC , Poma A . Molecularly imprinted polymers and electrospinning: manufacturing convergence for next‐level applications. Adv Funct Mater. 2020;30(32):2001955. 10.1002/adfm.202001955

[20] Poma A , Guerreiro A , Whitcombe MJ , Piletska EV , Turner APF , Piletsky SA . Solid‐phase synthesis of molecularly imprinted polymer nanoparticles with a reusable template—“plastic antibodies”. Adv Funct Mater. 2013;23(22):2821‐2827. 10.1002/adfm.201202397 26869870 PMC4746745

[21] Poma A , Guerreiro A , Caygill S , Moczko E , Piletsky S . Automatic reactor for solid‐phase synthesis of molecularly imprinted polymeric nanoparticles (MIP NPs) in water. RSC Adv. 2014;4(8):4203‐4206. 10.1039/C3RA46838K 26722622 PMC4693954

[22] Poma A , Brahmbhatt H , Pendergraff HM , Watts JK , Turner NW . Generation of novel hybrid aptamer‐molecularly imprinted polymeric nanoparticles. Adv Mater. 2015;27(4):750‐758. 10.1002/adma.201404235 25413444

[23] Poma A , Brahmbhatt H , Watts JK , Turner NW . Nucleoside‐tailored molecularly imprinted polymeric nanoparticles (MIP NPs). Macromolecules. 2014;47(18):6322‐6330. 10.1021/ma501530c

[24] Brahmbhatt H , Poma A , Pendergraff HM , Watts JK , Turner NW . Improvement of DNA recognition through molecular imprinting: hybrid oligomer imprinted polymeric nanoparticles (oligoMIP NPs). Biomater Sci. 2016;4(2):281‐287. 10.1039/c5bm00341e 26509192

[25] Subrahmanyam S , Guerreiro A , Poma A , Moczko E , Piletska E , Piletsky S . Optimisation of experimental conditions for synthesis of high affinity MIP nanoparticles. Eur Polym J. 2013;49(1):100‐105. 10.1016/j.eurpolymj.2012.09.022

[26] Muzyka K , Karim K , Guerreiro A , Poma A , Piletsky S . Optimisation of the synthesis of vancomycin‐selective molecularly imprinted polymer nanoparticles using automatic photoreactor. Nanoscale Res Lett. 2014;9(1):154. 10.1186/1556-276X-9-154 24685151 PMC3978078

[27] Guerreiro A , Poma A , Karim K , et al. Influence of surface‐imprinted nanoparticles on trypsin activity. Adv Healthcare Mater. 2014;3(9):1426‐1429. 10.1002/adhm.201300634 PMC4681057 24652761

[28] Bedwell TS , Anjum N , Ma Y , et al. New protocol for optimisation of polymer composition for imprinting of peptides and proteins. RSC Adv. 2019;9(48):27849‐27855. 10.1039/c9ra05009d 35530457 PMC9070758

[29] Ellis E , Zhang K , Lin Q , et al. Biocompatible pH‐responsive nanoparticles with a core‐anchored multilayer shell of triblock copolymers for enhanced cancer therapy. J Mater Chem B. 2017;5(23):4421‐4425. 10.1039/c7tb00654c 32263969

[30] Rodríguez‐Arco L , Poma A , Ruiz‐Pérez L , Scarpa E , Ngamkham K , Battaglia G . Molecular bionics ‐ engineering biomaterials at the molecular level using biological principles. Biomaterials. 2019;192:26‐50. 10.1016/j.biomaterials.2018.10.044 30419394

[31] Oz UC , Bolat ZB , Poma A , et al. Prostate cancer cell‐specific BikDDA delivery by targeted polymersomes. Appl Nanosci. 2020;10(9):3389‐3401. 10.1007/s13204-020-01287-0

[32] Kim ES , Kim D , Nyberg S , et al. LRP‐1 functionalized polymersomes enhance the efficacy of carnosine in experimental stroke. Sci Rep. 2020;10(1):699. 10.1038/s41598-020-57685-5 31959846 PMC6971073

[33] Liu M , Apriceno A , Sipin M , et al. Combinatorial entropy behaviour leads to range‐selective binding in ligand‐receptor interactions. Nat Commun. 2020;11(1):4836. 10.1038/s41467-020-18603-5 32973157 PMC7515919

[34] Gaitzsch J , Delahaye M , Poma A , Du Prez F , Battaglia G . Comparison of metal free polymer‐dye conjugation strategies in protic solvents. Polym Chem. 2016;7(17):3046‐3055. 10.1039/c6py00518g

[35] Malitesta C , Mazzotta E , Picca RA , Poma A , Chianella I , Piletsky SA . MIP sensors—the electrochemical approach. Anal Bioanal Chem. 2012;402(5):1827‐1846. 10.1007/s00216-011-5405-5 21947439

[36] Turner NW , Bramhmbhatt H , Szabo‐Vezse M , Poma A , Coker R , Piletsky SA . Analytical methods for determination of mycotoxins: an update (2009‐2014). Anal Chim Acta. 2015;901:12‐33. 10.1016/j.aca.2015.10.013 26614054

[37] Canfarotta F , Lezina L , Guerreiro A , et al. Specific drug delivery to cancer cells with double‐imprinted nanoparticles against epidermal growth factor receptor. Nano Lett. 2018;18(8):4641‐4646. 10.1021/acs.nanolett.7b03206 29969563

[38] Piletsky S , Canfarotta F , Poma A , Bossi AM , Piletsky S . Molecularly imprinted polymers for cell recognition. Trends Biotechnol. 2020;38(4):368‐387. 10.1016/j.tibtech.2019.10.002 31677857

[39] Liu R , Poma A . Advances in molecularly imprinted polymers as drug delivery systems. Molecules. 2021;26(12):3589. 10.3390/molecules26123589 34208380 PMC8231147

[40] Moczko E , Poma A , Guerreiro A , et al. Surface‐modified multifunctional MIP nanoparticles. Nanoscale. 2013;5(9):3733‐3741. 10.1039/c3nr00354j 23503559 PMC4724934

[41] Akgönüllü S , Kılıç S , Esen C , Denizli A . Molecularly imprinted polymer‐based sensors for protein detection. Polymers. 2023;15(3):629. 10.3390/polym15030629 36771930 PMC9919373

[42] Akgönüllü S , Denizli A . Molecular imprinting‐based sensors: lab‐on‐chip integration and biomedical applications. J Pharm Biomed Anal. 2023;225:115213. 10.1016/j.jpba.2022.115213 36621283

[43] Zhang H . Molecularly imprinted nanoparticles for biomedical applications. Adv Mater. 2020;32(3):1806328. 10.1002/adma.201806328 31090976

[44] Zi‐Hui M , Qin L . Determination of degradation products of nerve agents in human serum by solid phase extraction using molecularly imprinted polymer. Anal Chim Acta. 2001:435:121‐127.

[45] Le Moullec S , Bégos A , Pichon V , Bellier B . Selective extraction of organophosphorus nerve agent degradation products by molecularly imprinted solid‐phase extraction. J Chromatogr A. 2006;1108(1):7‐13. 10.1016/j.chroma.2005.12.105 16451804

[46] Mergola L , Scorrano S , Del Sole R , Lazzoi MR , Vasapollo G . Developments in the synthesis of a water compatible molecularly imprinted polymer as artificial receptor for detection of 3‐nitro‐l‐tyrosine in neurological diseases. Biosens Bioelectron. 2013;40(1):336‐341. 10.1016/j.bios.2012.07.074 22922080

[47] Rahmani A , Mohammadpour AH , Sahebnasagh A , Mohajeri SA . Haloperidol imprinted polymer: Preparation, evaluation, and application for drug assay in brain tissue. Anal Bioanal Chem. 2014;406(29):7729‐7739. 10.1007/s00216-014-8178-9 25258287

[48] Naklua W , Mahesh K , Chen YZ , Chen S , Roongnapa S . Molecularly imprinted polymer microprobes for manipulating neurological function by regulating temperature‐dependent molecular interactions. Process Biochem. 2016;51(1):142‐157. 10.1016/j.procbio.2015.11.002

[49] Habibi‐Khorasani M , Mohammadpour AH , Mohajeri SA . Development of solid‐phase microextraction coupled with liquid chromatography for analysis of tramadol in brain tissue using its molecularly imprinted polymer. Biomed Chromatogr. 2017;31(2):e3787. 10.1002/bmc.3787 27386837

[50] Luliński P , Bamburowicz‐Klimkowska M , Dana M , Maciejewska D . Development of a validated strategy for the determination of tryptamine in human cerebrospinal fluid in the presence of competitors using molecularly imprinted polymers. J Sep Sci. 2017;40(8):1824‐1833. 10.1002/jssc.201601349 28195396

[51] Roy KS , Mazumder A , Goud DR , Dubey DK . A simplistic designing of molecularly imprinted polymers for derivative of nerve agents marker using 31P{1H}NMR. Eur Polym J. 2018;98:105‐115. 10.1016/j.eurpolymj.2017.11.014

[52] Torab M , Jafari‐Sabet M , Najafizadeh P , Sadegipour A , Rahimi‐Moghaddam P , Ebrahimi SA . Oral administration of phenylalanine molecularly imprinted polymer (MIP) benefits PKU mouse model. J Inherit Metab Dis. 2022;45(4):696‐709. 10.1002/jimd.12513 35527480

[53] Huang BY , Chen YC , Wang GR , Liu CY . Preparation and evaluation of a monolithic molecularly imprinted polymer for the chiral separation of neurotransmitters and their analogues by capillary electrochromatography. J Chromatogr A. 2011;1218(6):849‐855. 10.1016/j.chroma.2010.12.054 21208621

[54] Jia M , Qin L , He XW , Li WY . Preparation and application of lysozyme imprinted monolithic column with dopamine as the functional monomer. J Mater Chem. 2012;22(2):707‐713. 10.1039/c1jm13134f

[55] Ji X , Li D , Li H . Preparation and application of a novel molecularly imprinted solid‐phase microextraction monolith for selective enrichment of cholecystokinin neuropeptides in human cerebrospinal fluid. Biomed Chromatogr. 2015;29(8):1280‐1289. 10.1002/bmc.3418 25616243

[56] Prathish K , Prasad K , Rao T , Suryanarayana M . Molecularly imprinted polymer‐based potentiometric sensor for degradation product of chemical warfare agents. Part I. Methylphosphonic acid. Talanta. 2007;71(5):1976‐1980. 10.1016/j.talanta.2006.09.002 19071551

[57] Jenkins AL , Manuel Uy O , Murray GM . Polymer‐based lanthanide luminescent sensors for the detection of nerve agents. Anal Commun. 1997;34(8):221‐224. 10.1039/a704220e 9949728

[58] Jenkins AL , Uy OM , Murray GM . Polymer‐based lanthanide luminescent sensor for detection of the hydrolysis product of the nerve agent soman in water. Anal Chem. 1999;71(2):373‐378. 10.1021/ac980985r 9949728

[59] Jenkins AL , Yin R , Jensen JL . Molecularly imprinted polymer sensors for pesticide and insecticide detection in water. Analyst (Lond). 2001;126(6):798‐802. 10.1039/b008853f 11445940

[60] Murray GM , Arnold BR , Kelly CA , Uy OM . Imprinted polymer sensors for contamination detection. Proc SPIE Int Soc Opt Eng. 2001;4206:131‐139. 10.1117/12.418722

[61] Southard GE , Van Houten KA , Ott EW , Murray GM . Luminescent sensing of organophosphates using europium(III) containing imprinted polymers prepared by RAFT polymerization. Anal Chim Acta. 2007;581(2):202‐207. 10.1016/j.aca.2006.08.027 17386445

[62] Wang C , Qi L , Liang R . A molecularly imprinted polymer‐based potentiometric sensor based on covalent recognition for the determination of dopamine. Anal Methods. 2021;13(5):620‐625. 10.1039/d0ay02100h 33480897

[63] Prasad BB , Srivastava S , Tiwari K , Sharma PS . Trace‐level sensing of dopamine in real samples using molecularly imprinted polymer‐sensor. Biochem Eng J. 2009;44(2‐3):232‐239. 10.1016/j.bej.2008.12.013

[64] Mintz Hemed N , Leal‐Ortiz S , Zhao ET , Melosh NA . On‐Demand, reversible, ultrasensitive polymer membrane based on molecular imprinting polymer. ACS Nano. 2023;17(6):5632‐5643. 10.1021/acsnano.2c11618 36913954 PMC10062346

[65] Mintz Hemed N , Hwang FJ , Zhao ET , Ding JB , Melosh NA . Multiplexed neurochemical sensing with sub‐nM sensitivity across 2.25 mm^2^ area. Biosens Bioelectron. 2024;261:261116474. 10.1016/j.bios.2024.116474 38870827

[66] Trotta F , Caldera F , Cavalli R , et al. Molecularly imprinted cyclodextrin nanosponges for the controlled delivery of L‐DOPA: perspectives for the treatment of Parkinson's disease. Expert Opin Drug Delivery. 2016;/12/01 2016 13(12):1671‐1680. 10.1080/17425247.2017.1248398 27737572

[67] Naklua W , Mahesh K , Aundorn P , et al. An imprinted dopamine receptor for discovery of highly potent and selective D3 analogues with neuroprotective effects. Process Biochem. 2015;50(10):1537‐1556. 10.1016/j.procbio.2015.06.018

[68] Pan J , Liu S , Jia H , et al. Rapid hydrolysis of nerve agent simulants by molecularly imprinted porous crosslinked polymer incorporating mononuclear zinc(II)‐picolinamine‐amidoxime module. J Catalysis. 2019;380:83‐90. 10.1016/j.jcat.2019.10.023

[69] Mohamed S , Balieu S , Petit E , et al. A versatile and recyclable molecularly imprinted polymer as an oxidative catalyst of sulfur derivatives: a new possible method for mustard gas and V nerve agent decontamination. Chem Commun. 2019;55(88):13243‐13246. 10.1039/c9cc04928b 31620710

[70] Zheng S , Pan J , Wang J , et al. Ag(I) pyridine‐amidoxime complex as the catalysis activity domain for the rapid hydrolysis of organothiophosphate‐based nerve agents: mechanistic evaluation and application. ACS Appl Mater Interfaces. 2021;13(29):34428‐34437. 10.1021/acsami.1c09003 34278774

[71] Cristallini C , Bellotti E , Spezia F , Rosellini E , Cascone MG , Barbani N . A new strategy to reduce amyloid deposition using peptide‐imprinted membranes. J Appl Biomater Biomech. 2016;14(2):129‐136. 10.5301/jabfm.5000288 27056481

[72] Nguyen PQH , Duong DD , Kwun JD , Lee NY . Hybrid elastomer–plastic microfluidic device as a convenient model for mimicking the blood–brain barrier in vitro. Biomed Microdevices. 2019;21(4):90. 10.1007/s10544-019-0446-1 31686217

[73] Osmani B , Schift H , Vogelsang K , et al. Hierarchically structured polydimethylsiloxane films for ultra‐soft neural interfaces. Micro Nano Eng. 2020;7:7100051. 10.1016/j.mne.2020.100051

[74] Prathish KP , Vishnuvardhan V , Rao TP . Rational design of in situ monolithic imprinted polymer membranes for the potentiometric sensing of diethyl chlorophosphate—a chemical warfare agent simulant. Electroanalysis. 2009;21(9):1048‐1056. 10.1002/elan.200804515

[75] DiCesare JC , Parker J , Horne SN , Kita J , Earni R , Peeples C . Progress in developing nerve agent sensors using combinatorial techniques. MRS Proceedings. 2003;787:G2.3. 10.1557/proc-787-g2.3

[76] Zhou Y , Yu B , Shiu E , Levon K . Potentiometric sensing of chemical warfare agents: surface imprinted polymer integrated with an indium tin oxide electrode. Anal Chem. 2004;76(10):2689‐2693. 10.1021/ac035072y 15144176

[77] Sezigen S , Kaya SI , Bakirhan NK , Ozkan SA . Development of a molecularly imprinted polymer‐based electrochemical sensor for the selective detection of nerve agent VX metabolite ethyl methylphosphonic acid in human plasma and urine samples. Anal Bioanal Chem. 2024;416:1505‐1515. 10.1007/s00216-024-05155-6 38267586 PMC10861733

[78] Chen PY , Nien PC , Ho KC . Highly Selective Dopamine Sensor based on an Imprinted SAM/Mediator Gold Electrode. 2009:285‐288.

[79] Chen PY , Nien PC , Wu CT , Wu TH , Lin CW , Ho KC . Fabrication of a molecularly imprinted polymer sensor by self‐assembling monolayer/mediator system. Anal Chim Acta. 2009;643(1‐2):38‐44. 10.1016/j.aca.2009.04.004 19446061

[80] Huang CY , Lee MH , Wu ZH , et al. A portable potentiostat with molecularly imprinted polymeric electrode for dopamine sensing. 2009.

[81] Lakshmi D , Bossi A , Whitcombe MJ , et al. Electrochemical sensor for catechol and dopamine based on a catalytic molecularly imprinted polymer‐conducting polymer hybrid recognition element. Anal Chem. 2009;81(9):3576‐3584. 10.1021/ac802536p 19354259

[82] Gómez‐Caballero A , Ugarte A , Sánchez‐Ortega A , Unceta N , Goicolea MA , Barrio RJ . Molecularly imprinted poly[tetra(o‐aminophenyl)porphyrin] as a stable and selective coating for the development of voltammetric sensors. J Electroanal Chem. 2010;638(2):246‐253. 10.1016/j.jelechem.2009.11.006

[83] Song W , Chen Y , Xu J , Yang XR , Tian DB . Dopamine sensor based on molecularly imprinted electrosynthesized polymers. 2010:1909‐1914.

[84] Dutta P , Pernites RB , Danda C , Advincula RC . SPR detection of dopamine using cathodically electropolymerized, molecularly imprinted poly‐p‐aminostyrene thin films. Macromol Chem Phys. 2011;212(22):2439‐2451. 10.1002/macp.201100365

[85] Prasad BB , Kumar D , Madhuri R , Tiwari MP . Sol‐gel derived multiwalled carbon nanotubes ceramic electrode modified with molecularly imprinted polymer for ultra trace sensing of dopamine in real samples. Electrochim Acta. 2011;56(20):7202‐7211. 10.1016/j.electacta.2011.04.090

[86] Vergara AV , Pernites RB , Pascua S , Binag CA , Advincula RC . QCM sensing of a chemical nerve agent analog via electropolymerized molecularly imprinted polythiophene films. J Polym Sci Part A Polym Chem. 2012;50(4):675‐685. 10.1002/pola.25077

[87] Zeng H , Wang D , Yu J . A molecule‐imprinted polyaniline membrane modified on carbon fiber for detection of glycine. Bio‐Med Mater Eng. 2014;24:1085‐1091. 10.3233/BME-130907 24212000

[88] Hiller T , Li LL , Holthoff EL , Bamieh B , Turner KL . System identification, design, and implementation of amplitude feedback control on a nonlinear parametric MEM resonator for trace nerve agent sensing. J Microelectromech Syst. 2015;24(5):1275‐1284. 7047694 10.1109/JMEMS.2015.2400398

[89] Li Y , Song H , Zhang L , et al. Supportless electrochemical sensor based on molecularly imprinted polymer modified nanoporous microrod for determination of dopamine at trace level. Biosens Bioelectron. 2016;78:308‐314. 10.1016/j.bios.2015.11.063 26630285

[90] Yang J , Hu Y , Li Y . Molecularly imprinted polymer‐decorated signal on‐off ratiometric electrochemical sensor for selective and robust dopamine detection. Biosens Bioelectron. 2019;135:224‐230. 10.1016/j.bios.2019.03.054 31030030

[91] Lee MH , Thomas JL , Su ZL , et al. Epitope imprinting of alpha‐synuclein for sensing in Parkinson's brain organoid culture medium. Biosens Bioelectron. 2021;175:175112852. 10.1016/j.bios.2020.112852 33288425

[92] Vais RD , Yadegari H , Heli H , Sattarahmady N. A β‐amyloid(1‐42) biosensor based on molecularly imprinted poly‐pyrrole for early diagnosis of alzheimer's disease. J Biomed Phys Eng. 2021;11(2):215‐228. 10.31661/jbpe.v0i0.1070 33937128 PMC8064131

[93] Yuan X , Gao X , Yuan Y , Ji Y , Xiong Z , Zhao L . Fe3O4/graphene molecularly imprinted composite for selective separation of catecholamine neurotransmitters and their analysis in rat brain tissues. Talanta. 2021;224:224121843. 10.1016/j.talanta.2020.121843 33379061

[94] Dhinesh Kumar M , Karthikeyan M , Sharma N , et al. Molecular imprinting synthetic receptor based sensor for determination of Parkinson's disease biomarker DJ‐1. Microchem J. 2022;183:183107959. 10.1016/j.microc.2022.107959

[95] Mani A , Rajeev MR , Anirudhan TS . Silver decorated silanized graphene oxide based molecularly surface imprinted electrochemical sensor for the trace level detection of L‐tryptophan. Mater Chem Phys. 2023;299:299127445. 10.1016/j.matchemphys.2023.127445

[96] Dhanjai N , Yu N , Mugo SM . A flexible‐imprinted capacitive sensor for rapid detection of adrenaline. Talanta. 2019;204:602‐606. 10.1016/j.talanta.2019.06.016 31357341

[97] Emam S , Nasrollahpour M , Colarusso B , et al. Detection of presymptomatic Alzheimer's disease through breath biomarkers. Alzheimer's Dement (Amsterdam, Netherlands). 2020;12(1):12088. 10.1002/dad2.12088 PMC7560498 33088894

[98] Lee MH , Liu KT , Thomas JL , et al. Peptide‐imprinted poly(hydroxymethyl 3,4‐ethylenedioxythiophene) nanotubes for detection of α synuclein in human brain organoids. ACS Appl Nano Mater. 2020;3(8):8027‐8036. 10.1021/acsanm.0c01476

[99] Pereira MV , Marques AC , Oliveira D , et al. Paper‐based platform with an in situ molecularly imprinted polymer for β‐amyloid. ACS Omega. 2020;5(21):12057‐12066. 10.1021/acsomega.0c00062 32548384 PMC7271027

[100] Liu H , Zhang C , Wang C , et al. A highly selective and sensitive sensor for promethazine based on molecularly imprinted interface coated Au/Sn bimetal nanoclusters functionalized acupuncture needle microelectrode. Anal Chim Acta. 2023;1269:1269341395. 10.1016/j.aca.2023.341395 37290856

[101] Srivastava J , Kushwaha A , Singh M . Imprinted graphene‐starch nanocomposite matrix‐anchored EQCM platform for highly selective sensing of epinephrine. NANO. 2018;13(11):1850131. 10.1142/S179329201850131X

[102] Lin CY , Tsai SH , Tai DF . Detection of oxytocin, atrial natriuretic peptide, and brain natriuretic peptide using novel imprinted polymers produced with amphiphilic monomers. J Pept Sci. 2019;25(3):e3150. 10.1002/psc.3150 30723971

[103] Parnianchi F , Kashanian S , Nazari M , Peacock M , Omidfar K , Varmira K . Ultrasensitive electrochemical sensor based on molecular imprinted polymer and ferromagnetic nanocomposite for bilirubin analysis in the saliva and serum of newborns. Microchem J. 2022;179:179107474. 10.1016/j.microc.2022.107474

[104] Gupta N , Shah K , Singh M . An epitope‐imprinted piezoelectric diagnostic tool for *Neisseria meningitidis* detection. J Mol Recognit. 2016;29(12):572‐579. 10.1002/jmr.2557 27488811

[105] Gupta N , Singh RS , Shah K , Prasad R , Singh M . Epitope imprinting of iron binding protein of *Neisseria meningitidis* bacteria through multiple monomers imprinting approach. J Mol Recognit. 2018;31(7):e2709. 10.1002/jmr.2709 29630761

[106] Altintas Z , Takiden A , Utesch T , et al. Integrated approaches toward high‐affinity artificial protein binders obtained via computationally simulated epitopes for protein recognition. Adv Funct Mater. 2019;29(15):1807332. 10.1002/adfm.201807332

[107] Ayankojo AG , Boroznjak R , Reut J , Tuvikene J , Timmusk T , Syritski V . Electrochemical sensor based on molecularly imprinted polymer for rapid quantitative detection of brain‐derived neurotrophic factor. Sens Actuators B. 2023;397:397134656. 10.1016/j.snb.2023.134656

[108] Ma X , Li M , Tong P , Zhao C , Li J , Xu G . A strategy for construction of highly sensitive glycosyl imprinted electrochemical sensor based on sandwich‐like multiple signal enhancement and determination of neural cell adhesion molecule. Biosens Bioelectron. 2020;156:156112150. 10.1016/j.bios.2020.112150 32275575

[109] Pathak A , Gupta BD . Ultra‐selective fiber optic SPR platform for the sensing of dopamine in synthetic cerebrospinal fluid incorporating permselective nafion membrane and surface imprinted MWCNTs‐PPy matrix. Biosens Bioelectron. 2019;133:205‐214. 10.1016/j.bios.2019.03.023 30939397

[110] Disley J , Gil‐Ramírez G , Gonzalez‐Rodriguez J . Chitosan‐based molecularly imprinted polymers for effective trapping of the nerve agent simulant dimethyl methylphosphonate. ACS Appl Polym Mater. 2023;5(1):935‐942. 10.1021/acsapm.2c01859

[111] Jiang P , Niu Y , Cao J , Xie D , Li J , Guo T . A MOF‐doped molecularly imprinted polymer/MOF hybrid gel incorporating with pH‐buffering sodium acrylate for practical detoxification of organophosphorus nerve agents. Chem Eng J. 2024;481:481148377. 10.1016/j.cej.2023.148377

[112] Bărăian A‐I , Iacob B‐C , Sorițău O , et al. Ruxolitinib‐loaded imprinted polymeric drug reservoir for the local management of post‐surgical residual glioblastoma cells. Polymers. 2023;15(4):965. 10.3390/polym15040965 36850247 PMC9962605

[113] Say R , Erdem M , Ersöz A , Türk H , Denizli A . Biomimetic catalysis of an organophosphate by molecularly surface imprinted polymers. Appl Catal, A. 2005;286(2):221‐225. 10.1016/j.apcata.2005.03.015

[114] Wang P , Zhang A , Jin Y , et al. Molecularly imprinted layer‐coated hollow polysaccharide microcapsules toward gate‐controlled release of water‐soluble drugs. RSC Adv. 2014;4(50):26063‐26073. 10.1039/c4ra04444d

[115] Gu X , Huang J , Zhang L , et al. Efficient discovery and capture of new neuronal nitric oxide synthase–postsynaptic density protein‐95 uncouplers from herbal medicines using magnetic molecularly imprinted polymers as artificial antibodies. J Sep Sci. 2017;40(17):3522‐3534. 10.1002/jssc.201700595 28704580

[116] Huang J , Sun C , Yao D , et al. Novel surface imprinted magnetic mesoporous silica as artificial antibodies for efficient discovery and capture of candidate nNOS‐PSD‐95 uncouplers for stroke treatment. J Mater Chem B. 2018;6(10):1531‐1542. 10.1039/c7tb03044d 32254217

[117] Zhu S , Wang X , Zheng Z , Zhao XE , Bai Y , Liu H . Synchronous measuring of triptolide changes in rat brain and blood and its application to a comparative pharmacokinetic study in normal and Alzheimer's disease rats. J Pharm Biomed Anal. 2020;185:185113263. 10.1016/j.jpba.2020.113263 32203895

[118] Gao S , Sun L , Zhou X , Zhu S , Liu H , Zhao XE . Simultaneous and dynamic measurement of Schisandrol A changes in rat blood and brain and its comparative pharmacokinetic study in control and Parkinson's disease rats by dual‐probe in vivo microdialysis. J Chromatogr A. 2023;1695:1695463950. 10.1016/j.chroma.2023.463950 37003077

[119] Malosse L , Buvat P , Adès D , Siove A . Detection of degradation products of chemical warfare agents by highly porous molecularly imprinted microspheres. Analyst (Lond). 2008;133(5):588‐595. 10.1039/b713713c 18427678

[120] Liu QS , Liu R , He HM , Cui J , Pang ZR , Yin XY . Preparation and application of picroside II molecularly imprinted ploymer for TCM researching. Adv Mater Res. 2011;287‐290:1987‐1990. 10.4028/www.scientific.net/AMR.287-290.1987

[121] Mao Y , Bao Y , Gan S , Li F , Niu L . Electrochemical sensor for dopamine based on a novel graphene‐molecular imprinted polymers composite recognition element. Biosens Bioelectron. 2011;28(1):291‐297. 10.1016/j.bios.2011.07.034 21824760

[122] Aaryashree J , Choudhary AK , Yoshimi Y . Disposable sensor chips with molecularly imprinted carbon paste electrodes for monitoring anti‐epileptic drugs. Sensors. 2023;23(6):3271. 10.3390/s23063271 36991982 PMC10059048

[123] BelBruno JJ . Molecularly imprinted polymers. Chem Rev. 2019;119(1):94‐119. 10.1021/acs.chemrev.8b00171 30246529

[124] Ma X , Tian Y , Yang R , et al. Nanotechnology In healthcare, and its safety and environmental risks. J Nanobiotechnol. 2024;/11/15 2024 22(1):715. 10.1186/s12951-024-02901-x PMC11566612 39548502

[125] Ma X , Knowles JC , Poma A . Biodegradable and sustainable synthetic antibodies—a perspective. Pharmaceutics. 2023;15(5):1440. 10.3390/pharmaceutics15051440 37242682 PMC10222044

[126] Akhoundian M , Alizadeh T , Pan G . Fabrication of the enzyme‐less voltammetric bilirubin sensor based on sol‐gel imprinted polymer. Electroanalysis. 2020;32(3):479‐488. 10.1002/elan.201900410

[127] Mandani S , Rezaei B , Ensafi AA . Sensitive imprinted optical sensor based on mesoporous structure and green nanoparticles for the detection of methamphetamine in plasma and urine. Spectrochim Acta, Part A. 2020;231:231118077. 10.1016/j.saa.2020.118077 32007904

[128] Kassem S , Piletsky SS , Yesilkaya H , et al. Assessing the in vivo biocompatibility of molecularly imprinted polymer nanoparticles. Polymers. 2022;14(21):4582. 10.3390/polym14214582 36365575 PMC9655879

[129] Amiri A , Faridbod F , Zoughi S . Selective and rapid optical detection of citalopram using a fluorescent probe based on carbon quantum dots embedded in silica molecularly imprinted polymer. J Fluorescence. 2024;34(3):1171‐1181. 10.1007/s10895-023-03323-y 37493859

[130] Fallah‐Darrehchi M , Zahedi P , Safarian S , Ghaffari‐Bohlouli P , Aeinehvand R . Conductive conduit based on electrospun poly (L‐lactide‐Co‐D, L‐lactide) nanofibers containing 4‐aminopyridine‐loaded molecularly imprinted poly (methacrylic acid) nanoparticles used for peripheral nerve regeneration. Int J Biiol Macromol. 2021;190:499‐507. 10.1016/j.ijbiomac.2021.09.009 34499956

[131] Liu L , Grillo F , Canfarotta F , et al. Carboxyl‐fentanyl detection using optical fibre grating‐based sensors functionalised with molecularly imprinted nanoparticles. Biosens Bioelectron. 2021;177:177113002. 10.1016/j.bios.2021.113002 33486137

[132] Asadi E , Abdouss M , Leblanc RM , Ezzati N , Wilson JN , Kordestani D . Synthesis, characterization and in vivo drug delivery study of a biodegradable nano‐structured molecularly imprinted polymer based on cross‐linker of fructose. Polymer. 2016;97:226‐237. 10.1016/j.polymer.2016.05.031

[133] Hashemi‐Moghaddam H , Kashi M , Mowla SJ , Nouraee N . Separation of microRNA 21 as a cancer marker from glioblastoma cell line using molecularly imprinted polymer coated on silica nanoparticles. J Sep Sci. 2016;39(18):3564‐3570. 10.1002/jssc.201600736 27422098

[134] Yao D , Zhang L , Huang J , et al. A surface magnetic imprinted polymers as artificial receptors for selective and efficient capturing of new neuronal nitric oxide synthase–post synaptic density protein‐95 uncouplers. J Pharm Biomed Anal. 2018;154:180‐190. 10.1016/j.jpba.2018.03.003 29550707

[135] Lee MH , Jan JS , Thomas JL , et al. Cellular therapy using Epitope‐Imprinted composite nanoparticles to remove α‐synuclein from an in vitro model. Cells. 2022;11(16):2584. 10.3390/cells11162584 36010659 PMC9406856

[136] Zhang W , Zhang L , Sun S , Wang B , Jiang L , Zhu L . An effective and simple strategy for highly selective and anti‐interference detection of dopamine based on the carbon quantum dots‐molecularly imprinted polymers modified electrode. Microchem J. 2023;195:195109340. 10.1016/j.microc.2023.109340

[137] Yağmuroğlu O . Molecularly imprinted polymer‐based potentiometric sensor for the selective and sensitive detection of nerve agent simulant parathion. Def Sci J. 2022;72(3):343‐352. 10.14429/dsj.72.17625

[138] Canfarotta F , Poma A , Guerreiro A , Piletsky S . Solid‐phase synthesis of molecularly imprinted nanoparticles. Nat Protoc. 2016;/03/01 2016 11(3):443‐455. 10.1038/nprot.2016.030 26866789

[139] Cowen T , Bedwell TS , Piletska EV , Rice H , Piletsky SA . Nanoparticle‐induced enhancement of cholinesterase activity in the presence of malathion: a potential nerve agent therapeutic. Int J Pharm. 2022;629:629122406. 10.1016/j.ijpharm.2022.122406 36395924

[140] Yoshimi Y , Katsumata Y , Osawa N , Ogishita N , Kadoya R . Synthesis of fluorescent molecularly imprinted polymer nanoparticles sensing small neurotransmitters with high selectivity using immobilized templates with regulated surface density. Nanomaterials. 2023;13(1):212. 10.3390/nano13010212 36616121 PMC9824157

[141] Zhang Z , Ma L , Yuan H , Chen Z , Lv Y . Solid‐phase screening and synthesis of molecularly imprinted nanoparticles for selective recognition and detection of brain natriuretic peptide. Adv Healthcare Mater. 2023;12(13):2300146. 10.1002/adhm.202300146 36737673

[142] Hou T , Zhang N , Yan C , et al. Curcumin‐loaded protein imprinted mesoporous nanosphere for inhibiting amyloid aggregation. Int J Biiol Macromol. 2022;221:334‐345. 10.1016/j.ijbiomac.2022.08.185 36084870

[143] Pan L , Ding Y , Ni X , et al. Modeling rapid and selective capture of nNOS‐PSD‐95 uncouplers from Sanhuang Xiexin decoction by novel molecularly imprinted polymers based on metal‐organic frameworks. RSC Adv. 2020;10(13):7671‐7681. 10.1039/c9ra10537a 35492204 PMC9049783

[144] Afshar EA , Taher MA , Karimi F , Karaman C , Moradi O . Ultrasensitive and highly selective “turn‐on” fluorescent sensor for the detection and measurement of melatonin in juice samples. Chemosphere. 2022;295:295133869. 10.1016/j.chemosphere.2022.133869 35134401

[145] Bektaşoğlu E , Özkütük EB , Ersöz A , Say R . Development of new molecular imprinted solid phase extraction material for dimethoate. Spectrosc Lett. 2014;47(3):168‐176. 10.1080/00387010.2013.774285

[146] Jorge AR , Chernobryva M , Rigby SEJ , Watkinson M , Resmini M . Incorporation of Cobalt‐Cyclen complexes into templated nanogels results in enhanced activity. Chem A Eur J. 2016;22(11):3764‐3774. 10.1002/chem.201503946 PMC4797703 26661923

[147] Figueiredo EC , Seabra CL , Mendes TV , et al. Molecularly imprinted nanoparticles as drug carriers to the brain. J Mater Sci. 2023;58(46):17578‐17593. 10.1007/s10853-023-09053-7

[148] Kan X , Zhao Y , Geng Z , Wang Z , Zhu JJ . Composites of multiwalled carbon nanotubes and molecularly imprinted polymers for dopamine recognition. J Phys Chem C. 2008;112(13):4849‐4854. 10.1021/jp077445v

[149] Boyd JW , Cobb GP , Southard GE , Murray GM . Development of molecularly imprinted polymer sensors for chemical warfare agents. Johns Hopkins APL Tech Dig. 2004;25(1):44‐49.

[150] Ugalde GA , de Bairros AV . Determination of diethyl chlorophosphate for the recognition of organophosphorus chemical warfare agents. Sensing of Deadly Toxic Chemical Warfare Agents, Nerve Agent Simulants, and their Toxicological Aspects. Elsevier; 2022:97‐109.

[151] Disley J , Gil‐Ramirez G , Gonzalez‐Rodriguez J. A computational study to produce a Molecular Imprinted Polymer for gas sensing of the nerve agent simulant Dimethyl Methylphosphonate. SPIE; 2023:

[152] John H , Worek F , Thiermann H . LC‐MS‐based procedures for monitoring of toxic organophosphorus compounds and verification of pesticide and nerve agent poisoning. Anal Bioanal Chem. 2008;391(1):97‐116. 10.1007/s00216-008-1925-z 18330546

[153] Aleksenko SS . Liquid chromatography with mass‐spectrometric detection for the determination of chemical warfare agents and their degradation products. J Anal Chem (Transl of Zh Anal Khim). 2012;67(2):82‐97. 10.1134/S1061934812020025

[154] Lu W , Xue M , Xu Z , et al. Molecularly imprinted polymers for the sensing of explosives and chemical warfare agents. Curr Org Chem. 2015;19(1):62‐71. 10.2174/1385272819666141201215551

[155] Mazuryk J , Klepacka K , Kutner W , Sharma PS . Glyphosate separating and sensing for precision agriculture and environmental protection in the era of smart materials. Environ Sci Technol. 2023;57(27):9898‐9924. 10.1021/acs.est.3c01269 37384557 PMC10339735

[156] Garcinuño RM , Chianella I , Guerreiro A , et al. The stabilisation of receptor structure in low cross‐linked MIPs by an immobilised template. Soft Matter. 2009;5(2):311‐317. 10.1039/b804476g

[157] Mwanza C , Zhang WZ , Mulenga K , Ding SN . Advancing green chemistry in environmental monitoring: the role of electropolymerized molecularly imprinted polymer‐based electrochemical sensors. Green Chem. 2024;26(23):11490‐11517. 10.1039/d4gc03250k

[158] Toh JE , Lee CS , Lim WH , Pichika MR , Chua BW . Stimulus‐responsive MOF‐hydrogel composites: classification, preparation, characterization, and their advancement in medical treatments. Open Chem. 2024;22(1):20240061. 10.1515/chem-2024-0061

[159] Azzouz A , Goud KY , Raza N , et al. Nanomaterial‐based electrochemical sensors for the detection of neurochemicals in biological matrices. Trends Anal Chem. 2019;110:15‐34. 10.1016/j.trac.2018.08.002

[160] Elugoke SE , Adekunle AS , Fayemi OE , et al. Molecularly imprinted polymers (MIPs) based electrochemical sensors for the determination of catecholamine neurotransmitters—Review. Electrochem Sci Adv. 2021;1(2):e2000026. 10.1002/elsa.202000026

[161] Zhang Y , Jiang N , Yetisen AK . Brain neurochemical monitoring. Biosens Bioelectron. 2021;161:189113351. 10.1016/j.bios.2021.113351 34049083

[162] Engelborghs S , Marescau B , De Deyn PP . Amino acids and biogenic amines in cerebrospinal fluid of patients with Parkinson's disease. Neurochem Res. 2003;28(8):1145‐1150. 10.1023/A:1024255208563 12834252

[163] Wightman RM , May LJ , Michael AC . Detection of dopamine dynamics in the brain. Anal Chem. 1988;60(13):769A‐779A. 10.1021/ac00164a001 3063135

[164] Liu R , Li J , Zhong T , Long L . Graphene modified molecular imprinting electrochemical sensor for determining the content of dopamine. Curr Anal Chem. 2019;15(6):628‐634. 10.2174/1573411014666180730112304

[165] Gu S , Wang W , Wang F , Huang JH . Neuromodulator and emotion biomarker for stress induced mental disorders. Neural Plast. 2016;2016(1):2609128. 10.1155/2016/2609128 27051536 PMC4808661

[166] Martinez M , Frank A , Diez‐Tejedor E , Hernanz A . Amino acid concentrations in cerebrospinal fluid and serum in Alzheimer's disease and vascular dementia. J Neural Transm Park Dis Dement Sect. 1993;6(1):1‐9. 10.1007/BF02252617 8216758

[167] Emam S , Nasrollahpour M , Colarusso B , et al. Detection of presymptomatic Alzheimer's disease through breath biomarkers. Alzheimer's Dement (Amsterdam, Netherlands). 2020;12(1):12088. 10.1002/dad2.12088 PMC7560498 33088894

[168] Perez‐Grijalba V , Romero J , Pesini P , et al. Plasma Abeta42/40 ratio detects early stages of Alzheimer's disease and correlates with CSF and neuroimaging biomarkers in the AB255 study. J Prev Alzheimers Dis. 2019;6(1):34‐41. 10.14283/jpad.2018.41 30569084 PMC12280714

[169] Bayer TA , Wirths O . Intracellular accumulation of amyloid‐beta—a predictor for synaptic dysfunction and neuron loss in Alzheimer's disease. Front Aging Neurosci. 2010;2:8. 10.3389/fnagi.2010.00008 20552046 PMC2879032

[170] Miranda M , Morici JF , Zanoni MB , Bekinschtein P . Brain‐derived neurotrophic factor: a key molecule for memory in the healthy and the pathological brain. Front Cell Neurosci. 2019;13:363. 10.3389/fncel.2019.00363 31440144 PMC6692714

[171] Wu Y , Du X , Yang R , et al. Association between depressive symptoms and serum brain‐derived neurotrophic factor levels in patients with first‐episode and Drug‐Naïve Schizophrenia. Front Psychiatry. 2022;13:911384. 10.3389/fpsyt.2022.911384 35757201 PMC9218218

[172] Geleta GS . Recent advances in electrochemical sensors based on molecularly imprinted polymers and nanomaterials for detection of ascorbic acid, dopamine, and uric acid: A review. Sensing and Bio‐Sensing Research. 2024;43:100610. 10.1016/j.sbsr.2023.100610

[173] Mao SS , Zhou XQ , Qin XF , Xu XJ , Xie MH . Preparation of magnetic nanomolecularly imprinted polymers and their application in investigation of trace PQQ‐DA products in organisms. J Instrum Anal. 2019;38(4):482‐487. 10.3969/j.issn.1004-4957.2019.04.018

[174] Claude B , Nehmé R , Morin P . Analysis of urinary neurotransmitters by capillary electrophoresis: sensitivity enhancement using field‐amplified sample injection and molecular imprinted polymer solid phase extraction. Anal Chim Acta. 2011;699(2):242‐248. 10.1016/j.aca.2011.05.014 21704780

[175] Butterfield DA , Reed TT , Perluigi M , et al. Elevated levels of 3‐nitrotyrosine in brain from subjects with amnestic mild cognitive impairment: implications for the role of nitration in the progression of Alzheimer's disease. Brain Res. 2007;1148:243‐248. 10.1016/j.brainres.2007.02.084 17395167 PMC1934617

[176] Mihm MJ , Schanbacher BL , Wallace BL , Wallace LJ , Uretsky NJ , Bauer JA . Free 3‐nitrotyrosine causes striatal neurodegeneration in vivo. J Neurosci. 2001;21(11):RC149. 10.1523/JNEUROSCI.21-11-j0003.2001 11344255 PMC6762709

[177] Blanchard‐Fillion B , Prou D , Polydoro M , et al. Metabolism of 3‐nitrotyrosine induces apoptotic death in dopaminergic cells. J Neurosci. 2006;26(23):6124‐6130. 10.1523/JNEUROSCI.1038-06.2006 16763020 PMC6675196

[178] Zucchi R , Chiellini G , Scanlan TS , Grandy DK . Trace amine‐associated receptors and their ligands. Br J Pharmacol. 2006;149(8):967‐978. 10.1038/sj.bjp.0706948 17088868 PMC2014643

[179] Broadley KJ . The vascular effects of trace amines and amphetamines. Pharmacol Ther. 2010;125(3):363‐375. 10.1016/j.pharmthera.2009.11.005 19948186

[180] Young SN , Gauthier S , Anderson GM , Purdy WC . Tryptophan, 5‐hydroxyindoleacetic acid and indoleacetic acid in human cerebrospinal fluid: interrelationships and the influence of age, sex, epilepsy and anticonvulsant drugs. J Neurol Neurosurg Psychiatry. 1980;43(5):438‐445. 10.1136/jnnp.43.5.438 6158559 PMC490572

[181] Claude B , Morin P , Denoroy L . Selective solid‐phase extraction of catecholamines and metanephrines from serum using a new molecularly imprinted polymer. J Liq Chromatogr Relat Technol. 2014;37(18):2624‐2638. 10.1080/10826076.2013.853310

[182] Zhou WH , Tang SF , Yao QH , Chen FR , Yang HH , Wang XR . A quartz crystal microbalance sensor based on mussel‐inspired molecularly imprinted polymer. Biosens Bioelectron. 2010;26(2):585‐589. 10.1016/j.bios.2010.07.024 20685108

[183] Zhou WH , Lu CH , Guo XC , Chen FR , Yang HH , Wang XR . Mussel‐inspired molecularly imprinted polymer coating superparamagnetic nanoparticles for protein recognition. J Mater Chem. 2010;20(5):880‐883. 10.1039/b916619j

[184] Chen X , Ostovan A , Arabi M , Wang Y , Chen L , Li J . Molecular imprinting‐based SERS detection strategy for the large‐size protein quantitation and curbing non‐specific recognition. Anal Chem. 2024;96(16):6417‐6425. 10.1021/acs.analchem.4c00541 38606984

[185] Arabi M , Ostovan A , Wang Y , et al. Chiral molecular imprinting‐based SERS detection strategy for absolute enantiomeric discrimination. Nat Commun. 2022;13(1):5757. 10.1038/s41467-022-33448-w 36180485 PMC9525700

[186] Arabi M , Ostovan A , Zhang Z , et al. Label‐free SERS detection of Raman‐Inactive protein biomarkers by Raman reporter indicator: toward ultrasensitivity and universality. Biosens Bioelectron. 2021;174:112825. 10.1016/j.bios.2020.112825 33243696

[187] Palladino P , Bettazzi F , Scarano S . Polydopamine: surface coating, molecular imprinting, and electrochemistry‐successful applications and future perspectives in (bio)analysis. Anal Bioanal Chem. 2019;411(19):4327‐4338. 10.1007/s00216-019-01665-w 30806753

[188] Clegg JR , Wechsler ME , Peppas NA . Vision for functionally decorated and molecularly imprinted polymers in regenerative engineering. Regen Eng Transl Med. 2017;3(3):166‐175. 10.1007/s40883-017-0028-9 30906848 PMC6426136

[189] Bărăian AI , Iacob BC , Bodoki AE , Bodoki E . In vivo applications of molecularly imprinted polymers for drug delivery: a pharmaceutical perspective. Int J Mol Sci. 2022;23(22):14071. 10.3390/ijms232214071 36430548 PMC9698206

[190] Wang X , Wang YH , Liu S , Li C . Construction of the brain‐targeting drug carrier through imprinting of nicotinic acetylcholine receptor α7. Yao xue xue bao = Acta pharmaceutica Sinica. 2017;52(3):488‐493. 10.16438/j.0513-4870.2016-1188 29979864

[191] Gladding M , Shen X , Snyder MP , Havel PJ , Adams SH . Interindividual variability in postprandial plasma fructose patterns in adults. Nutrients. 2024;16(18):3079. 39339679 10.3390/nu16183079 PMC11435096

[192] Wang Y , Zhao T , Dai P , Jiang N , Li F . Employment of molecularly imprinted polymers to high‐throughput screen nNOS‐PSD‐95 interruptions: structure and dynamics investigations on monomer‐template complexation. Chemphyschem. 2016;17(6):893‐901. 10.1002/cphc.201500941 26728445

[193] He HL , Pan LL , Gu XL , et al. Efficient discovery and capturing of nNOS‐PSD‐95 uncouplers from *Trifolium pratense* . Zhongguo Zhong yao za zhi = Zhongguo zhongyao zazhi = China journal of Chinese materia medica. 2018;43(4):748‐754. 29600650 10.19540/j.cnki.cjcmm.20171208.001

[194] Lamaoui A , Palacios‐Santander JM , Amine A , Cubillana‐Aguilera L . Molecularly imprinted polymers based on polydopamine: assessment of non‐specific adsorption. Microchem J. 2021;164:106043. 10.1016/j.microc.2021.106043

[195] Han W , Han X , Liu Z , et al. Facile modification of protein‐imprinted polydopamine coatings over nanoparticles with enhanced binding selectivity. Chem Eng J. 2020;385:123463. 10.1016/j.cej.2019.123463

[196] Mattiassi S , Rizwan M , Grigsby CL , Zaw AM , Leong KW , Yim EKF . Enhanced efficiency of nonviral direct neuronal reprogramming on topographical patterns. Biomater Sci. 2021;9(15):5175‐5191. 10.1039/d1bm00400j 34128504

[197] Kincses A , Vigh JP , Petrovszki D , et al. The use of sensors in blood‐brain barrier‐on‐a‐chip devices: current practice and future directions. Review Biosensors. 2023;13(3):357. 10.3390/bios13030357 36979569 PMC10046513

[198] Yang R , Ma X , Xuan M , et al. Advances in molecularly imprinted polymers for bone biomarker detection and therapeutic applications. ChemistryOpen. 2025;14:e2500127. 10.1002/open.202500127 PMC12598817 40677115

[199] Ma X , Allahou LW , Yang R , et al. Antiviral molecularly imprinted polymers: engineered precision for multifunctional therapeutic strategies. Mater Sci Eng R Rep. 2026;167:101099. 10.1016/j.mser.2025.101099

[200] Arabi M , Ostovan A , Li J , et al. Molecular imprinting: green perspectives and strategies. Adv Mater. 2021;33(30):2100543. 10.1002/adma.202100543 34145950

[201] Hubrecht RC , Carter E . The 3Rs and humane experimental technique: implementing change. Animals. 2019;9(10):754. 10.3390/ani9100754 31575048 PMC6826930

[202] Ma X , Poma A . 10 ‐ Clinical translation and envisioned impact of nanotech for infection control: economy, government policy and public awareness. In: Poma A , Rizzello L , eds. Nanotechnology Tools for Infection Control. Elsevier; 2025:299‐392.

[203] Suzaei FM , Daryanavard SM , Abdel‐Rehim A , Bassyouni F , Abdel‐Rehim M . Recent molecularly imprinted polymers applications in bioanalysis. Chem Zvesti. 2023;77(2):619‐655. 10.1007/s11696-022-02488-3 36213319 PMC9524737

[204] Refaat D , Aggour MG , Farghali AA , et al. Strategies for molecular imprinting and the evolution of MIP nanoparticles As plastic Antibodies‐Synthesis and applications. Int J Mol Sci. 2019;20(24):6304. 10.3390/ijms20246304 31847152 PMC6940816

[205] Tlili A , Attia G , Khaoulani S , et al. Contribution to the understanding of the interaction between a polydopamine molecular imprint and a protein model: ionic strength and pH effect investigation. Sensors. 2021;21(2):619. 10.3390/s21020619 33477338 PMC7830185

[206] Kumar R , Shafique MS , Chapa SOM , Madou MJ . Recent advances in MOF‐based materials for biosensing applications. Sensors. 2025;25(8):2473. 10.3390/s25082473 40285162 PMC12031313

[207] Yarahmadi B , Hashemianzadeh SM , Milani Hosseini SM‐R. Machine‐learning‐based predictions of imprinting quality using ensemble and non‐linear regression algorithms. Sci Rep. 2023;13(1):12111. 10.1038/s41598-023-39374-1 37495673 PMC10372080

